# Radiotherapy resistance driven by Asparagine endopeptidase through ATR pathway modulation in breast cancer

**DOI:** 10.1186/s13046-025-03334-6

**Published:** 2025-02-27

**Authors:** Macarena Morillo-Huesca, Ignacio G. López-Cepero, Ryan Conesa-Bakkali, Mercedes Tomé, Colin Watts, Pablo Huertas, Gema Moreno-Bueno, Raúl V. Durán, Jonathan Martínez-Fábregas

**Affiliations:** 1 Centro Andaluz de Biología Molecular y Medicina Regenerativa - CABIMER, Consejo Superior de Investigaciones Científicas (CSIC), Universidad de Sevilla, Universidad Pablo de Olavide, Américo Vespucio 24, Seville, 41092 Spain; 2 Departamento de Bioquímica Vegetal y Biología Molecular, Facultad de Biología, Universidad de Sevilla, Avenida Reina Mercedes, Seville, 41012 Spain; 3 Division of Cell Signalling and Immunology, School of Life Sciences, University of Dundee, Dundee, DD1 5EH UK; 4 Departamento de Genética, Facultad de Biología, Universidad de Sevilla, Avenida Reina Mercedes, Seville, 41012 Spain; 5 Instituto de Investigaciones Biomédicas Sols-Morreale (CSIC-UAM), C/ Arturo Duperier 4, Madrid, 28029 Spain; 6 Centro de Investigación Biomédica en Red de Cáncer (CIBERONC), Instituto de Salud Carlos III, Madrid, Spain; 7 Fundación MD Anderson Internacional, C/ Gómez Hemans 1, Madrid, 28033 Spain; 8 Translational Cancer Research Group. Area 3 Cancer, Instituto Ramón y Cajal de Investigación Sanitaria (IRYCIS), Madrid, Spain

## Abstract

**Background:**

Tumor resistance represents a major challenge in the current oncology landscape. Asparagine endopeptidase (AEP) overexpression correlates with worse prognosis and reduced overall survival in most human solid tumors. However, the underlying mechanisms of the connection between AEP and reduced overall survival in cancer patients remain unclear.

**Methods:**

High-throughput proteomics, cellular and molecular biology approaches and clinical data from breast cancer (BC) patients were used to identify novel, biologically relevant AEP targets. Immunoblotting and qPCR analyses were used to quantify protein and mRNA levels. Flow cytometry, confocal microscopy, chemical inhibitors, siRNA- and shRNA-silencing and DNA repair assays were used as functional assays. In-silico analyses using the TCGA BC dataset and immunofluorescence assays in an independent cohort of invasive ductal (ID) BC patients were used to validate the clinical relevance of our findings.

**Results:**

Here we showed a dual role for AEP in genomic stability and radiotherapy resistance in BC patients by suppressing ATR and PPP1R10 levels. Reduced ATR and PPP1R10 levels were found in BC patients expressing high AEP levels and correlated with worst prognosis. Mechanistically, AEP suppresses ATR levels, reducing DNA damage-induced cell death, and PPP1R10 levels, promoting Chek1/P53 cell cycle checkpoint activation, allowing BC cells to efficiently repair DNA. Functional studies revealed AEP-deficiency results in genomic instability, increased DNA damage signaling, reduced Chek1/P53 activation, impaired DNA repair and cell death, with phosphatase inhibitors restoring the DNA damage response in AEP-deficient BC cells. Furthermore, AEP inhibition sensitized BC cells to the chemotherapeutic reagents cisplatin and etoposide. Immunofluorescence assays in an independent cohort of IDBC patients showed increased AEP levels in ductal cells. These analyses showed that higher AEP levels in radioresistant IDBC patients resulted in ATR nuclear eviction, revealing AEPhigh/ATRlow protein levels as an efficient predictive biomarker for the stratification of radioresistant patients.

**Conclusion:**

The newly identified AEP/ATR/PPP1R10 axis plays a dual role in genomic stability and radiotherapy resistance in BC. Our work provides new clues to the underlying mechanisms of tumor resistance and strong evidence validating the AEP/ATR axis as a novel predictive biomarker and therapeutic target for the stratification and treatment of radioresistant BC patients.

**Supplementary Information:**

The online version contains supplementary material available at 10.1186/s13046-025-03334-6.

## Background

Despite the contribution of technical advances in both chemotherapy and radiotherapy strategies to improvements in treatment outcomes and the quality of life of cancer patients [1], tumor resistance remains a significant challenge in the current oncology landscape. This is because 80–90% of deaths in cancer patients are associated to cancer resistance [2, 3], a complex phenomenon linked to a plethora of cellular alterations, including increased DNA repair, defects in the induction of apoptosis, and autophagy [1, 4]. Therefore, the identification of novel druggable players involved in resistance could allow us to improve cancer therapies by sensitizing cancer cells to current treatments.

Proteases are associated with tumor progression across cancer types. Although, initially considered to promote tumor invasion through extracellular matrix degradation, emerging evidence has revealed unexpected functions for these proteases during the onset and progression of cancer [5]. Unfortunately, the use of broad-range protease inhibitors in cancer therapy has failed to improve patient outcomes [6, 7]. Therefore, the identification of the relevant proteases associated with specific cancer types and the characterization of their molecular targets are paramount for their translation to the clinical setting. Among proteases, AEP is the only known protease that specifically hydrolyzes asparaginyl peptide bonds, and to a lesser extent, aspartyl peptide bonds [8], thus suggesting that AEP has specific regulatory functions rather than simple recycling functions.

Under pathological conditions, AEP has been shown to play key roles in the onset and progression of neurodegenerative diseases [9–14] and cancer [15–20]. In this context, the identification of its biological targets in neurodegenerative diseases, such as Parkinson´s and Alzheimer´s, has allowed to understand its role in the onset and progression of these diseases at the molecular level, thus revealing AEP as a potential therapeutic target for the treatment of these conditions [9–14]. However, even though the connection between AEP overexpression in a plethora of human solid tumors and poor prognosis, increased malignancy, and worse overall survival has been reported [21–27], the mechanistic insights allowing to rationalize its role in the onset and progression of this disease are still lacking, hampering our ability to design novel, improved strategies for cancer treatment.

Here, we demonstrate that AEP plays a dual role in the resistance of breast cancer (BC) patients to genotoxic stress through the regulation of ATR and PPP1R10 levels, key DNA repair effectors [28, 29]. Specifically, AEP by suppressing ATR levels in BC cells increases genotoxic stress tolerance and reduces DNA damage-induced apoptosis, while maintaining proper cell cycle checkpoints allowing for efficient DNA repair by reducing PPP1R10 and protein phosphatase activity. Conversely, AEP deficiency results in BC cell death characterized by increased genomic instability and DNA damage signaling. Remarkably, in silico analyses using available clinical data from BC patients and further validated using an independent array of samples from IDBC patients showed that patients with AEP^high^/ATR^low^ levels can be efficiently identified as nonresponder patients, thus reinforcing the clinical significance of our findings and revealing AEP as a key contributor to tumor resistance. Besides, we also show that AEP inhibition sensitizes BC cells to cisplatin and etoposide treatment, further supporting a novel role for AEP in genotoxic stress tolerance in cancer cells and revealing AEP as a potential novel therapeutic target for the treatment of radioresistant BC cancer patients. Finally, our findings delineate a pan-cancer rule revealing a similar relationship between AEP^high^/ATR^low^ levels and reduced overall survival in other types of cancer. In summary, our data revealed AEP as a key factor contributing to cancer resistance, providing the rationale for the design of new strategies to treat resistant tumors by combining AEP inhibitors with current chemo- and radiotherapy approaches to sensitize them to genotoxic stress.

## Results

### AEP activity is required for cell cycle progression and cell division in cancer cells

**Fig. 1:**
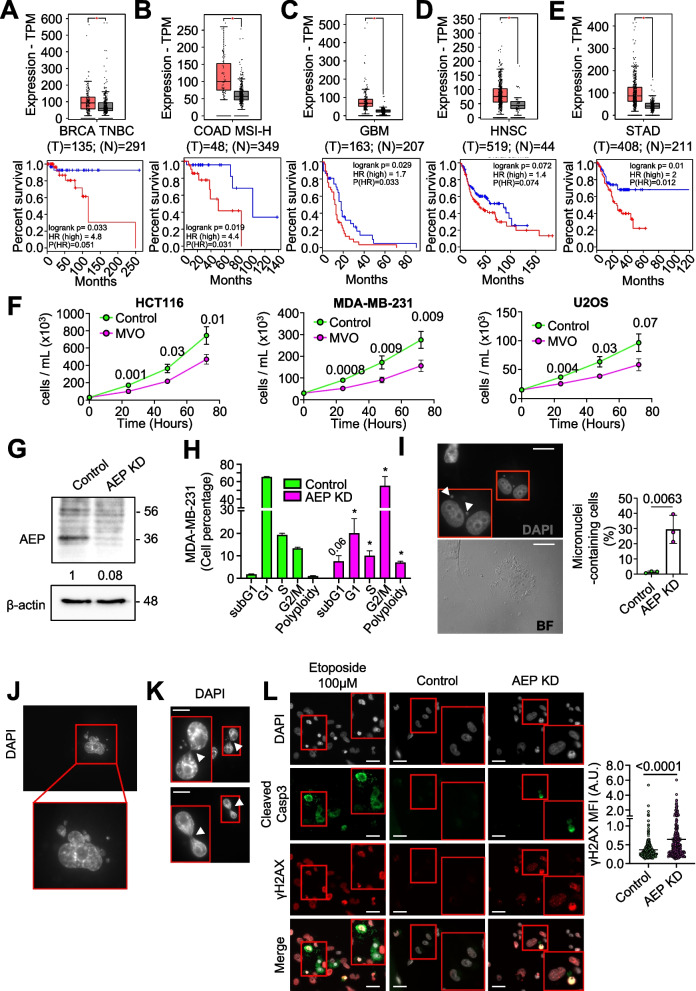
AEP deficiency reduces cell proliferation and impact cell cycle in cancer cells. Differential expression levels of AEP in different types of tumors were obtained using the GEPIA2 online application and extracted from The Cancer Genome Atlas (TCGA) database. Kaplan–Meier curves were constructed using the GEPIA2 online application to assess the correlation between AEP expression levels (low AEP blue line; high AEP red line) in different types of human tumors and overall survival. **A** AEP expression levels in BRCA triple-negative breast cancer (TNBC) patients (*n* = 135, red) as compared to normal samples (*n* = 291, grey) [upper panel] and Kaplan–Meier curves showing overall survival of patients with high (red line) or low (blue line) AEP expression levels [lower panel]. **B** AEP expression levels in colon adenocarcinoma with high genomic instability (COAD MSI-H) patients (*n* = 48, red) as compared to normal samples (*n* = 349, grey) [upper panel] and Kaplan–Meier curves showing overall survival of patients with high (red line) or low (blue line) AEP expression levels [lower panel]. **C** AEP expression levels in glioblastoma (GBM) patients (*n* = 163, red) as compared to normal samples (*n* = 207, grey) [upper panel] and Kaplan–Meier curves showing overall survival of patients with high (red line) or low (blue line) AEP expression levels [lower panel]. **D** AEP expression levels in head and neck squamous cell carcinoma (HNSC) patients (*n* = 519, red) as compared to normal samples (*n* = 44, grey) [upper panel] and Kaplan–Meier curves showing overall survival of patients with high (red line) or low (blue line) AEP expression levels [lower panel]. **E** AEP expression levels in stomach adenocarcinoma (STAD) patients (*n* = 408, red) as compared to normal samples (*n* = 211, grey) [upper panel] and Kaplan–Meier curves showing overall survival of patients with high (red line) or low (blue line) AEP expression levels [lower panel]. **F** Proliferation curves of different cancer cell lines (HCT116 [left panel], MDA-MB-231 [middle panel] and U2OS [right panel]) upon MVO-mediated AEP inhibition. Data represents average of 5 independent, biological replicas ± SD. **G** Immunoblot showing the shRNA-mediated AEP knock-down in MDA-MB-231 cells. **H** Cell cycle analyses of control and AEP shRNA-transduced MDA-MB-231 cells. Data represents average of 4 independent, biological replicas ± SD. * *p* value < 0. 01. **I** Micrographs showing the presence of micronuclei in MDA-MB-231 cells upon shRNA-mediated AEP KD alongside quantitation. Data represents the average of 3 independent experiments, each one including more than 100 cell ± SD. Size bar = 27 μm. **J** Micrographs showing examples of polyploid cells in shRNA-mediated AEP KD MDA-MB-231 cells. **K** Micrographs showing internuclear DNA bridges in shRNA-mediated AEP KD MDA-MB-231 cells. Arrowheads indicate DNA bridges. Size bar = 27 μm. **L** Micrographs showing γH2AX (red) and cleaved caspase 3 (green) staining in MDA-MB-231 cells treated with 100 μM etoposide for 24 h (left panels), MDA-MB-231 control cells (middle panels) and AEP shRNA-transduced MDA-MB-231 cells (right panels) alongside quantitation representing γH2AX mean fluorescence intensity (MFI) in cells negative for cleaved caspase 3. (n > 300 cells). Size bar = 27 μm

AEP is overexpressed and correlates with poor prognosis and reduced overall survival in many human solid tumors, such as breast cancer [17], colorectal cancer [19, 20], glioblastoma [30] and gastric cancer [31]. To explore this further we utilized the GEPIA2 online tool [32] and the TCGA database. These in silico analyses confirmed that AEP mRNA expression levels were significantly higher (*p* < 0.01) in these human solid tumors compared to normal samples (Fig. 1A-E, upper panels). Furthermore, survival analyses revealed that high AEP expression was statistically associated with poor prognosis and reduced overall survival in patients with these tumors (Fig. 1A-E, bottom panels). Remarkably, in triple negative BC, high AEP expression levels correlated with a great reduction in overall survival, whereas patients with low AEP expression levels had almost one hundred percent survival (Fig. 1A). For this reason, we decided to focus on understanding the role of AEP in BC.

Next, to characterize the role of AEP in cancer cells, we tested whether AEP inhibition with a highly specific inhibitor (MVO26630, hereafter referred to as MVO) [33] might affect cell proliferation. AEP inhibition significantly reduced cell proliferation in several cancer cell lines (Fig. 1F). As previously reported [34], AEP inhibition resulted in a compensatory increase in AEP levels in cancer cells aimed at the recovery of its activity (Supplementary Fig. 1A). Consequently, to inhibit this compensatory response, shRNA-mediated AEP knockdown (KD) approaches were used in MDA-MB-231 cells (Fig. 1G). AEP KD resulted in increased cell death and an expansion of the G2/M phase (Fig. 1H and Supplementary Fig. 1D). Notably, a significant increase in both the percentage of micronuclei-containing cells (Fig. 1I) and an increase in the number of polyploid cells (Fig. 1H and J and Supplementary Fig. 1D) in AEP KD MDA-MB-231 cells were observed, suggesting a potential role for AEP in suppressing genomic instability in BC cells. Additionally, AEP deficiency in cancer cells led to the formation of internuclear DNA bridges (Fig. 1K) which were positive for γH2AX (Supplementary Fig. 1I), consistent with an accumulation of DNA damage that led to chromosome segregation problems. Apoptosis can also result in γH2AX accumulation [35]. Therefore, to elucidate whether the increase in γH2AX observed in AEP KD MDA-MB-231 cells was due to genomic instability or to cell death, we decided to stain both control and AEP KD cells for γH2AX and the apoptotic marker cleaved caspase 3. 24-h treatment with 100 μM of the apoptosis inducer etoposide resulted in a significant increase in the number of apoptotic cells, as determined by positive staining for both γH2AX and cleaved caspase 3 (Fig. 1L, left panels). By contrast, AEP deficiency, even in the absence of external genotoxic insults, resulted in an increase in γH2AX compared to control cells, but not in cleaved caspase 3 levels, reflecting a lack of induction of apoptosis (Fig. 1L, middle and right panels and quantitation), and further reinforcing the role of AEP in genomic stability in BC cells. Similar results were obtained for U2OS cells upon AEP KD (Supplementary Fig. 1), further validating our observations in MDA-MB-231 cells. Taken together, these data suggest for the first time an unexpected role for AEP in chromosomal stability and DNA damage signaling/repair in cancer cells. However, the precise molecular mechanism (*i.e.*, the specific biological targets) by which AEP contributes to genomic stability in cancer remains unclear. Therefore, we undertook a high-throughput proteomic study to identify the biological targets and processes regulated by AEP, in order to gain insight into the mechanistic role of AEP in cancer cells.

### Chemical inhibition reveals noncanonical functions of AEP

As mentioned above, AEP inhibition frequently triggers a compensatory response [34, 36], making it difficult to identify potential AEP substrates. However, following a screening of different cell lines, we identified HEK293 cells as a cell line with high levels of AEP activity (Supplementary Fig. 2A). Next, we verified that 16-h MVO-treatment [33] effectively inhibited AEP activity in HEK293 cells without resulting in increased AEP activity (Supplementary Fig. 2B). These data revealed HEK293 cells as appropriate candidates for further experiments designed to identify novel AEP targets that could allow to understand its role in chromosomal stability/DNA damage response in cancer cells.

**Fig. 2:**
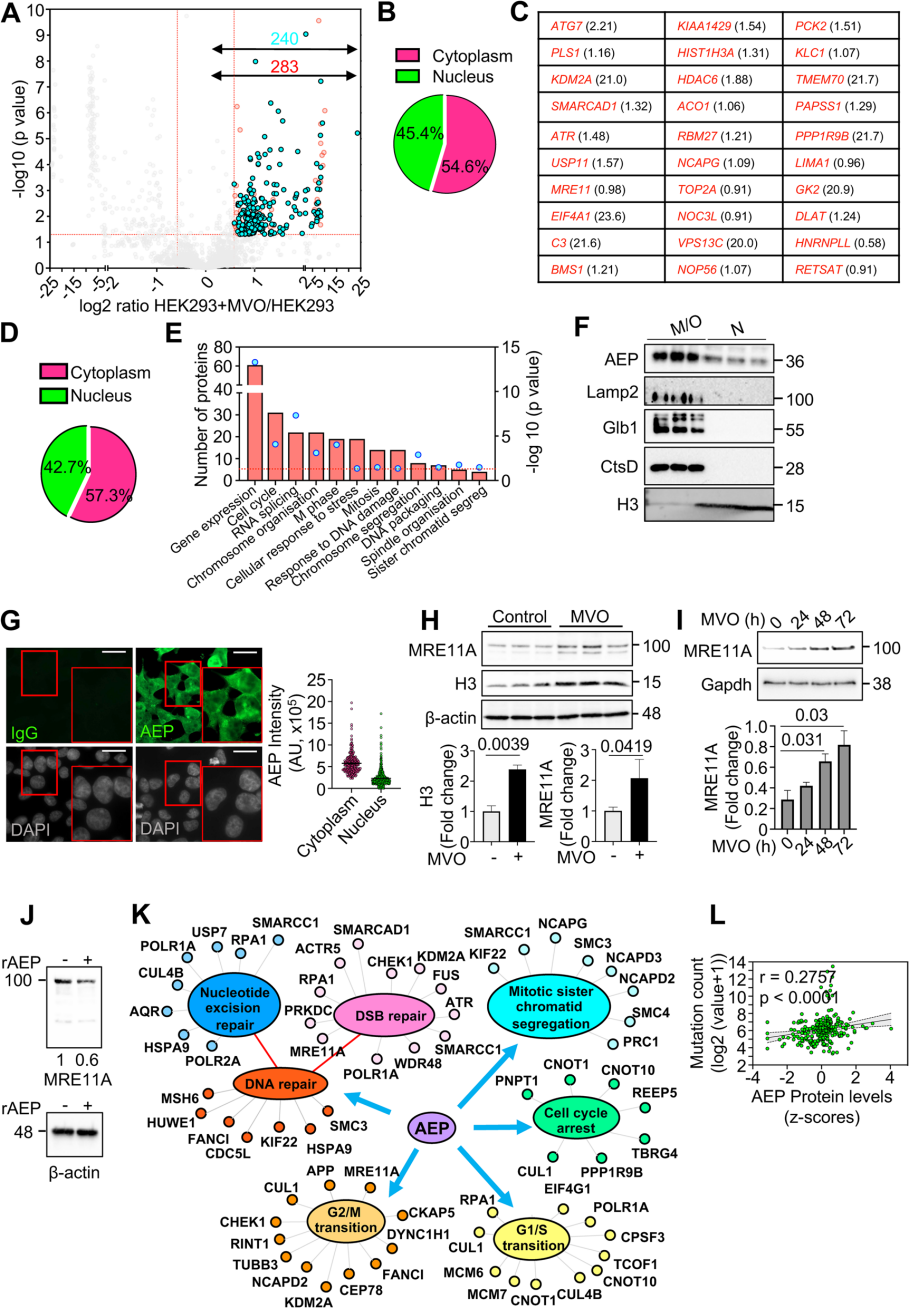
Proteomics study of MVO26630-mediated AEP inhibition in HEK293 cells reveals a putative nuclear role for AEP. **A** Volcano plot showing in red proteins whose levels are increased (283) and in cyan those that are increased and their putative cleavage site has been identified (240) upon 24-h MVO26630 treatment. **B** Pie chart showing nuclear and cytoplasmic distribution of the proteins accumulating upon MVO treatment. **C** Proteins showing the highest accumulation for which their putative AEP cleavage site has been identified upon MVO treatment as indicated by our proteomics data, alongside fold change in parenthesis (log2 of the HEK293 + MVO/HEK293 ratio). **D** Pie chart showing nuclear and cytoplasmic distribution of the proteins accumulating upon MVO treatment for which their putative AEP cleavage site has been identified. **E** GO analysis (Biological processes) of the proteins accumulating upon MVO treatment for which their putative AEP cleavage site has been identified (240 proteins). **F** Subcellular fractionation analyses (M/O – membrane/organelle, N—nucleus) of AEP localization in HEK293 cells, including Lamp2 as a lysosomal membrane marker, Glb1 and CtsD as soluble lysosomal hydrolases, and H3 as a nuclear marker. **G** Micrographs showing AEP nuclear localization in HEK293 cells under normal conditions alongside quantitation of the AEP intensity (n > 400 cells) corrected using a sheep IgG isotype, showing both cytoplasmic vs nuclear data (Size bar = 6um). **H** Immunoblot showing the levels of MRE11A and H3 in MVO-treated vs untreated HEK293 cells, alongside quantitation. Data represents the average of 3 independent, biological replicas ± SD. **I** Immunoblot showing the levels of MRE11A in HEK293 treated with MVO for 0, 24, 48 or 72 h, alongside quantitation. Data represents the average of 3 independent, biological replicas ± SD. **J** Immunoblot showing the in vitro digestion of MRE11A overexpressed in HEK293 cells using recombinant AEP (rAEP) in pH7.2 for 3 h. **K** Schematic representation of some of the newly identified targets of AEP highlighting the biological processes regulated by AEP as extracted from our GO analysis. **L** Correlation analyses between AEP protein expression levels (z-score) and mutation count (log2 (value + 1)) in TCGA pan cancer samples obtained through cBioportal

Accordingly, to further our knowledge of the relationship between AEP and genomic stability we used label-free, quantitative high-resolution mass spectrometry on untreated and 24-h MVO-treated HEK293 cells (Supplementary Fig. 2C). The combined analyses of our proteomic data yielded 3,766 identified proteins, of which 283 statistically accumulated upon AEP inhibition (Fig. 2A and Supplementary Table S1). The total number of peptides identified in the biological replicas was similar, with all replicates showing a Pearson correlation coefficient (r) value greater than 0.9 (Supplementary Figs. 2D and E). Finally, gene ontology (GO) analyses of the 283 proteins significantly accumulating upon AEP inhibition (Fig. 2A and C and Supplementary Table S1) revealed that 45.4% of these potential AEP targets localized to the nuclear compartment (Fig. 2B), thus suggesting a role for AEP in the regulation of nuclear targets.

### Identification of novel AEP cleavage sites

As previously described, AEP is the only known protease that specifically hydrolyzes asparaginyl and, to a lesser extent, aspartyl peptide bonds [8]. Thus, an additional analysis of our proteomic dataset was conducted, focusing on tryptic peptides that are statistically accumulated upon AEP inhibition. These peptides were required to contain asparagine (Asn) and/or aspartate (Asp) residues in their sequence, as they could represent specific AEP cleavage sites. These analyses yielded 1,323 Asn/Asp-containing peptides statistically overrepresented upon AEP inhibition (Supplementary Table S2), corresponding to 994 proteins. This approach enabled the identification of potential AEP cleavage sites for 240 out of the 283 proteins that accumulated upon AEP inhibition (Fig. 2A and Supplementary Tables S1-S2), thereby further reinforcing the potential role of AEP in the regulation of the levels of these proteins. Remarkably, some of the peptides identified through our proteomic analyses included already well-described AEP cleavage sites (Table 1 and Supplementary Table S2) [11, 37, 38], thus validating our experimental approach.

**Table 1:** Peptides identified in our proteomics approach containing previously identified AEP cleavage sites (highlighted in green) indicating the accumulation in MVO treated samples as log2 fold change and the p value

Peptide	Gene name	Uniprot ID	Log2 Fold change (Control/MVO)	P value	Reference
LQAALDDEEAGGRPAMEPGNGSLDLGGDSAGR	*HNRNPU*	Q00839	−0.727648	0.0115	Ziegler et al. 2024
ATGANATPLDFPSK	*SF1*	Q15637	−22.15507	6.01E-05	Ziegler et al. 2024
NYGILADATEQVGQHKDAYQVILDGVK	*API5*	Q9BZZ5	−22.679283	1.39E-05	Vidmar *et al. *2017
NWYIQATCATSGDGLYEGLDWLSNQLR	*ARF1*	P84077	−0.8937414	0.058	Vidmar et al. 2017
DGYQQNFK	*CAPRIN1*	Q14444	−21.1974	0.0001	Vidmar et al. 2017
AMGIMNSFVNDIFERIAGEASR	*HIST1H2BN*	U3KQK0	−0.6792178	0.02	Vidmar et al. 2017
GPAVGIDLGTTYSCVGVFQHGKVEIIANDQGNR	*HSPA8*	P11142	−20.312689	1.31E-05	Vidmar et al. 2017
VLGEAMTGISQNAK	*TLN1*	Q9Y490	−22.950227	2.31E-05	Vidmar et al. 2017
VEVERDNLAEDIMR	*VIM*	P08670	−1.5767547	0.035	Vidmar et al. 2017
TVETRDGQVINETSQHHDDLE	*VIM*	P08670	−1.5056379	0.056	Vidmar et al. 2017
ISIEMNGTLEDQLSHLK	*ACTN4*	O43707	−15.456436	0.098	Vidmar et al. 2017
LKTDNAGDQHGGGGGGGGGAGAAGGGGGGENYDDPHKTPASPVVHIR	*HNRNPL*	P14866	−0.972008	0.135	Vidmar et al. 2017
VESLEQEAANER	*APP*	H7C0V9	−13.94863	0.099	Zhang et al. 2015
EVVEEAENGR	*PTMA*	B8ZZQ6	−20.337713	9.09E-06	Sarandeses et al. 2003

Interestingly, more than 40% of the proteins that accumulated upon AEP inhibition and that contained putative AEP cleavage sites as defined above, were localized to the nuclear compartment (Fig. 2D). Moreover, a significant number of these proteins were associated with the DNA damage response, cell cycle regulation and mitosis/chromosome segregation (Fig. 2E). Therefore, our combined proteomic data further support the hypothesis that AEP has a physiological function in the regulation of key nuclear proteins and, importantly, provides potential targets of AEP to interrogate at the molecular level its role in the regulation of these biological processes in cancer (Fig. 1 and Supplementary Fig. 1). Next, we decided to examine the subcellular localization and activity of AEP in HEK293 cells. Subcellular fractionation assays confirmed that AEP was present both in the nuclear and in the membrane/organelle fractions of HEK293 cells. Proper fractionation was confirmed with Lamp2 [35] (a lysosomal membrane protein) and cathepsin D (CtsD) [39] and galactosidase beta 1 (Glb1) [40] (both, lysosomal soluble proteins) localizing exclusively to the membrane/organelle fraction, not in the nuclear fraction (Fig. 2F). Furthermore, confocal microscopy analyses (Fig. 2G) revealed that, while 69.91% ± 0.85 of AEP exhibited a clear cytoplasmic distribution, 30.09% ± 0.85 of AEP localized to the nuclear compartment. Finally, AEP activity in nuclear extracts that could be inhibited with MVO was detected using specific, fluorogenic substrates [34] in vitro (Supplementary Fig. 2F). These results confirm the nuclear localization and activity of AEP and raise the possibility that a nuclear pool of AEP directly regulates the levels of these proteins.

Finally, we sought to validate our proteomic data by determining whether AEP inhibition resulted in the accumulation of targets identified through our proteomic approach. Some of the potential AEP targets were related proteins containing highly conserved potential cleavage sites (Supplementary Fig. 2G). Recent reports have demonstrated the nuclear pH is similar to the cytoplasmic pH, varying from 7.0 to 7.3 [41–45]. Thus, to test the potential role of AEP in the degradation of these proteins, we performed in vitro digestion assays using recombinant AEP at pH7.2 to mimic extralysosomal, nuclear conditions, as previously reported [41–45]. In this experimental setting, AEP was able to completely digest GFP-tagged versions of a number of these proteins, including MCM6, MCM7, NOP56, NOP58, HSP90AA1 and HSP90AB1 (Supplementary Fig. 2H). As negative control, COXIV, a protein that was not identified as potential AEP target in our proteomics experiment (Supplementary Table 1), was resistant to AEP digestion (Supplementary Fig. 2H), confirming the specificity of AEP substrates. Furthermore, AEP inhibition led to the accumulation of MRE11A and H3 (Fig. 2H), both of which are among the highest ranked proteins identified as potential AEP targets in our proteomic approach (Fig. 2C). Long-term inhibition of AEP for 0, 24, 48 or 72 h in HEK293 cells resulted in the gradual accumulation of MRE11A, further validating our proteomics data (Fig. 2I). Finally, in vitro assays revealed that recombinant AEP can digest overexpressed MRE11A in pH7.2, thus mimicking the nuclear pH [41–45], with β-actin being resistant to AEP proteolytic activity (Fig. 2J), thus reinforcing the idea that these proteins are bona-fide AEP targets. Thus, our combined data validate a potential role for AEP in regulating DNA damage signaling and genomic stability through the fine-tuning of the levels of these key proteins (Fig. 2K). Notably, pan-cancer data analyses from the TCGA database (*n* = 263, including samples from breast, colorectal and ovarian epithelial cancers) revealed a positive correlation between tumor mutation count and the protein levels of AEP, further supporting a potential link between AEP and genomic stability (Fig. 2L) and revealing the clinical significance of our findings.

### AEP activity controls ATR levels and negatively correlates with ATR in breast cancer patient survival

**Fig. 3:**
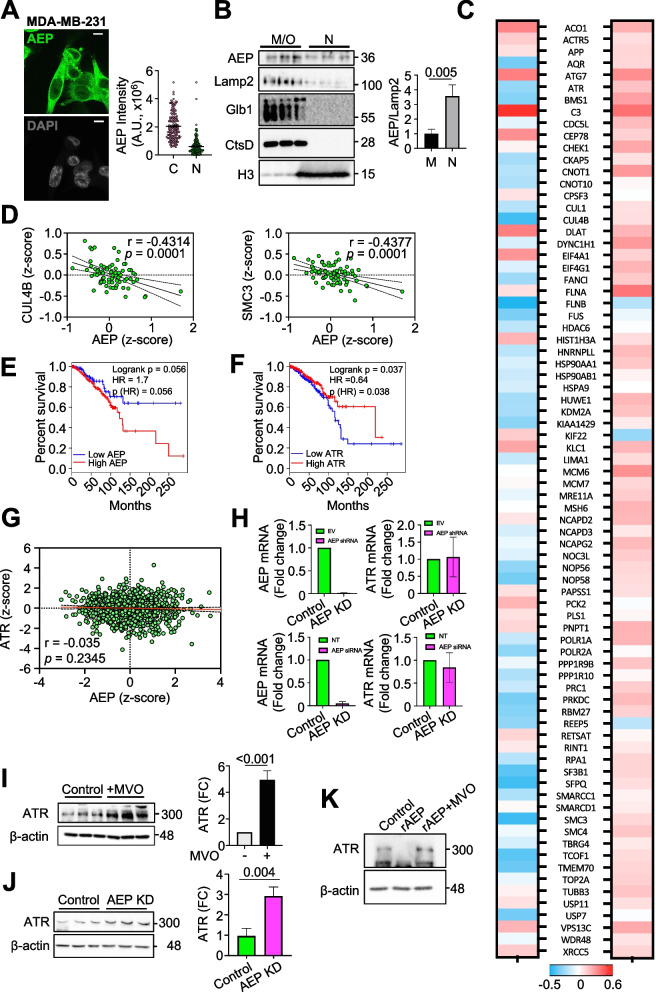
AEP localizes in the nuclear compartment of cancer cells and regulates the levels of ATR. **A** Immunofluorescence showing the subcellular localization of AEP in MDA-MB-231 (n > 150 cells) alongside quantitation showing the cytoplasmic (C) and nuclear (N) intensity, corrected using a sheep IgG antibody. (Size bar = 6um). **B** Immunoblot showing the nuclear (N) and membrane/organelle (M/O) localization of AEP in MDA-MB-231, including Lamp2 as a lysosomal membrane marker, Glb1 and CtsD as soluble lysosomal hydrolases and H3 as a nuclear marker, alongside quantitation. Data represents the average ± SD of three independent, biological replicas. **C** Correlation analyses between AEP and the main proteins identified as novel AEP targets in our proteomic experiment in breast cancer patients at the protein (left panel) and RNA (right panel) levels. **D** Correlation analysis of the protein expression levels of some of the novel AEP targets identified in our proteomic analyses (CUL4B and SMC3) vs AEP in BC patients using data obtained from the TCGA database. **E** Kaplan–Meier analysis of breast cancer patients expressing high (red line, *n* = 503) or low (blue line, *n* = 129) AEP levels. **F** Kaplan–Meier analysis of breast cancer patients expressing high (red line, *n* = 319) or low (blue line, *n* = 268) ATR levels. **G** Correlation analysis of the mRNA expression levels of ATR vs AEP in BC patients using data obtained from the TCGA database. **H** qPCR analyses of the mRNA expression levels of AEP and ATR in control (EV, empty vector) and AEP shRNA (upper panels) or control (NT, non-targeting) and AEP siRNA KD (bottom panels) MDA-MB-231 cells. **I** Immunoblot showing the effect of MVO-mediated AEP inhibition in the levels of ATR in MDA-MB-231 cells alongside quantitation. Data represents the average ± SD of three independent, biological replicas. **J** Immunoblot showing the effect of shRNA-mediated AEP knock-down in MDA-MB-231 cells in the levels of ATR alongside quantitation. Data represents the average ± SD of three independent, biological replicas. **K** Immunoblot showing the in vitro digestion of ATR using recombinantly expressed AEP at pH7.2 for 3 h

So far, our data obtained via both AEP chemical inhibition and genetic ablation suggest that AEP plays a role in cell cycle regulation, genomic stability, and DNA damage signaling in cancer cells (Fig. 1). Furthermore, our proteomic approach strongly supports these observations by elucidating a previously unidentified function of AEP as a regulator of the levels of key nuclear proteins involved in the regulation of these biological processes in HEK293 (Fig. 2). Therefore, to interrogate this new role of AEP in cancer cells, we analyzed its subcellular localization in BC cells. Both, confocal microscopy of endogenous AEP (Fig. 3A) and subcellular fractionation analyses (Fig. 3B) confirmed the nuclear localization of AEP in MDA-MB-231 cells. Remarkably, AEP is restricted to the cytoplasmic compartment in primary cells [34, 36], thus suggesting a pathological function for AEP in the nuclear compartment of cancer cells. Interestingly, similar results were obtained for colon carcinoma, osteosarcoma and glioblastoma cancer cell lines, further supporting the nuclear localization of AEP in cancer cells (Supplementary Figs. 3A and B).

Next, we wanted to test whether the novel AEP targets identified in our proteomic approach were relevant in BC patients. To this end, correlation analyses using the TCGA BRCA dataset between the levels of expression of AEP and some of the proteins identified in our proteomic study as AEP targets and involved in DNA damage signaling and cell cycle (Fig. 2, Supplementary Fig. 2 and Supplementary Table S1) were carried out. Remarkably, increased AEP protein levels were found to be associated with a decrease in most of these proteins (Fig. 3C, left panel), including key regulators of DNA damage signaling and cell cycle (Fig. 3D and Supplementary Fig. 3C), thus further supporting our proteomic data. Importantly, a similar correlation was not observed at the transcriptional level (Fig. 3C, right panel), thus confirming a direct role for AEP in the regulation of the levels of these proteins not only in cell lines, but also in BC patients, further supporting our hypothesis of a novel, unexpected role of AEP in the regulation of genomic stability and DNA damage in BC.

As shown above, high AEP expression levels correlated with reduced overall patient survival in different types of cancer (Fig. 1A-E), with AEP deficiency resulting in increased genomic instability, elevated γH2AX levels, and cell death (Fig. 1H-L). Furthermore, as shown in the proteomic data (Fig. 2 and Supplementary Table S1), AEP deficiency resulted in the accumulation of ATR, with reduced ATR protein levels observed in patients expressing high AEP protein levels (Fig. 3C, left panel), further supporting a biologically relevant role for AEP in regulating ATR levels. Interestingly, ATR is one of the kinases involved in the phosphorylation of H2AX in response to DNA damage [46–48]. Therefore, we wondered whether AEP and ATR correlated with overall survival in BC patients including all the subtypes (TNBC, luminal A, luminal B and Her2 +). Indeed, our analyses revealed that patients with high AEP expression levels had a poor prognosis and reduced overall survival (Fig. 3E), whereas the opposite was observed for ATR, with low ATR levels correlating with reduced survival (Fig. 3F). Remarkably, a similar observation was made in other types of cancer: high AEP expression levels (Supplementary Fig. 3D, upper panels) and low ATR levels (Supplementary Fig. 3D, bottom panels) correlated with poor prognosis and reduced overall survival, suggesting a more general role of the AEP/ATR axis in cancer.

Our analyses in BC patients showed a connection between AEP and ATR at the protein level (Fig. 3C, left panel), that could not be observed at the transcriptional level (Fig. 3C, right panel and Fig. 3G). Moreover, qPCR analyses revealed that AEP KD in MDA-MB-231 cells, which was achieved via the use of either specific shRNAs (upper panels) or siRNAs (lower panels) against AEP, did not affect ATR expression levels (Fig. 3H). These findings further strengthen the idea that AEP directly regulates ATR. Therefore, to further validate this connection we decided to study the effect of suppressing AEP in the levels of ATR in BC cells. Pharmacological inhibition and genetic ablation of AEP (Fig. 3I and J, respectively) resulted in a strong ATR accumulation in MDA-MB-231 cells, with similar results obtained in U2OS cells (Supplementary Fig. 3E), further validating a role for AEP in the regulation of ATR levels. In agreement with the finding that ATR is an AEP target, recombinant AEP was able to degrade in vitro purified ATR at pH7.2, with β-actin being resistant to AEP digestion, further reinforcing a role for nuclear AEP in the regulation of ATR levels (Fig. 3K). Finally, to substantiate our conclusion, we decided to assess the co-localization of AEP and ATR within the nuclear compartment of MDA-MB-231 cells. Proximity ligation assays confirmed the nuclear co-localization of AEP and ATR in MDA-MB-231 cells (Supplementary Fig. 3F), reinforcing a direct interaction between a nuclear pool of AEP and ATR within the nucleus of BC cells. Overall, our data demonstrated AEP as a direct regulator of ATR protein levels in BC patients, thus identifying ATR as the molecular target for understanding the role of AEP as a regulator of DNA damage signaling and genomic stability in BC cells.

### AEP contributes to breast cancer resistance to genotoxic stress by reducing DNA damage signaling through the regulation of ATR and PPP1R10 levels

**Fig. 4:**
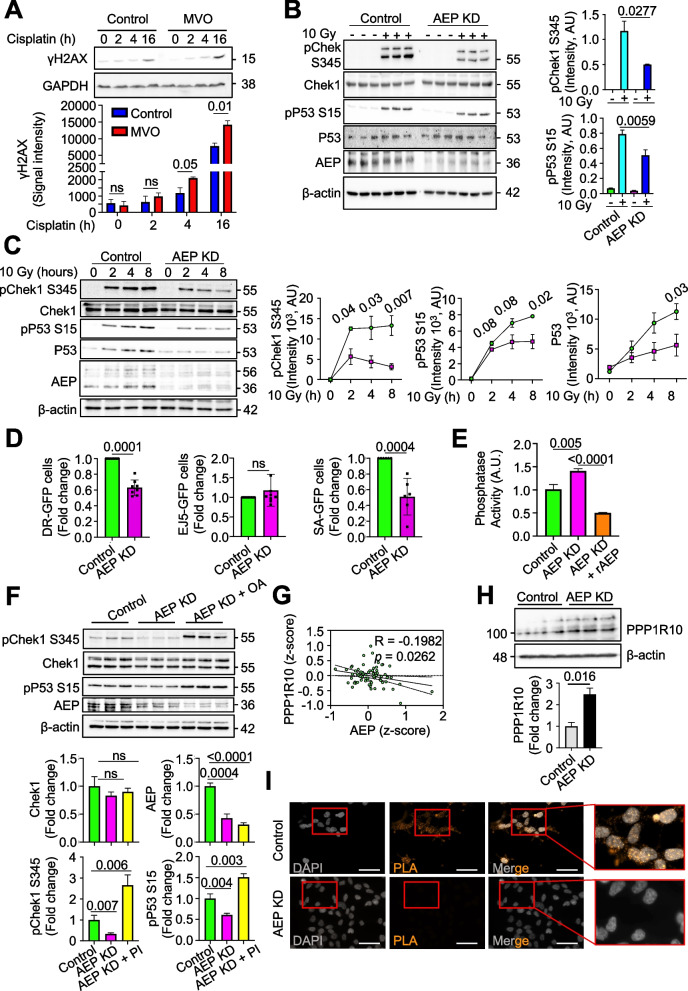
Nuclear AEP activity reduces DNA damage signaling while targeting phosphatase activity to maintain proper cell cycle checkpoint activation in breast cancer cells. **A** Immunoblot of γH2AX in MDA-MB-231 treated or not with MVO 50 μM for 48 h and then stimulated with cisplatin 3 μM, alongside quantitation. Data represents the mean + SD of three, independent biological replicas. **B** Immunoblot showing the phosphorylation levels of Chek1 and P53 upon 10 Gy radiation and 2 h recovery in both control and AEP KD MDA-MB-231, alongside quantitation. Data represents the mean ± SD of three independent, biological replicas. **C** Time course showing the phosphorylation levels pf Chek1 and P53 upon 10 Gy radiation followed by 2-, 4- or 8-h recovery, alongside quantitation in control (green circles) and AEP KD (pink squares) MDA-MB-231 cells. Data represents the mean ± SD of three independent, biological replicas. **D** Graph showing the effect of AEP deficiency in DNA repair using U2OS cells depleted or not of AEP and stably expressing DR-GFP, EJ5-GFP or SSA-GFP reporters constructs. After 72 h GFP fluorescence was analysed by flow cytometry. Data show average fold change ± SD of the number of positive cells for each system compared to control cells. Data was generated from at least six independent, biological replicas. **E** Phosphatase activity in control vs AEP KD ± rAEP MDA-MB-231 cells. Data represents the mean ± SD of three independent, biological replicas. **F** Immunoblot showing the levels of phosphorylation of Chek1 upon 10 Gy radiation and 2 h recovery in control MDA-MB-231 cells compared to AEP KD MDA-MB-231 pre-treated or not with 10 μM okadaic acid (OA) 30 min prior to irradiation, alongside quantitation. Data represents the mean ± SD of three independent, biological replicas. **G** Correlation analysis of the protein expression levels of PPP1R10 vs AEP in BC patients using data obtained from the TCGA database. **H** Immunoblot showing PPP1R10 levels in control and AEP KD MDA-MB-231 cells, alongside quantitation. Data represents the mean ± SD of three independent, biological replicas. **I** Proximity ligation assay (PLA) using anti-AEP and anti-PPP1R10 in control and AEP KD MDA-MB-231 cells

To advance our understanding of the AEP-ATR axis in BC at the molecular level, we explored the role of AEP deficiency in DNA damage signaling. First, we wondered whether the high levels of ATR observed in MDA-MB-231 cells upon AEP inhibition led to increased DNA damage signaling in response to genotoxic insults. Indeed, acute AEP inhibition resulted in increased DNA damage signaling in response to cisplatin treatment (Fig. 4A), confirming the role of AEP in DNA damage signaling by reducing H2AX phosphorylation (γH2AX) through the regulation of ATR levels (Figs. 2C, 3C, I-K).

In response to genotoxic insults, ATR phosphorylates Chek1 at serine 345, and subsequently pChek1 phosphorylates P53 at serine 15, thus inducing cell cycle arrest and allowing cells to repair DNA [49, 50]. Therefore, on the basis of our previous results and to further validate the connection between AEP and ATR in DNA damage signaling and chromosome stability, we hypothesized that the increase in ATR levels in AEP-deficient cancer cells (Figs. 2C, 3C, I-K and Supplementary Fig. 3E) could lead to increased pChek1 and pP53 levels, thus explaining the G2/M arrest observed in AEP-deficient cancer cells (Fig. 1H). However, AEP deficiency in MDA-MB-231 cells resulted in reduced phosphorylation of both pChek1-S345 and pP53-S15 after 2 h of recovery from a single dose of irradiation (10 Gy) (Fig. 4B). To resolve this, we tested whether there were differences in the kinetics of phosphorylation and dephosphorylation of both pChek1 and pP53. Interestingly, upon irradiation, AEP-deficient MDA-MB-231 cells presented reduced levels of pChek1 and increased pChek1 dephosphorylation rates (Fig. 4C, immunoblots and left panel), resulting in diminished levels of pP53 and total P53 accumulation (Fig. 4C, immunoblots and middle and right panels).

Thus, AEP deficiency in cancer cells resulted in faster dephosphorylation rates of both Chek1 and P53 (Fig. 4C) despite elevated ATR levels (Fig. 3I-K). Remarkably, this unexpected result allowed to rationalize a previous observation. AEP deficiency in cancer cells resulted in the accumulation of polyploid and micronuclei-containing cells, internuclear DNA bridges and elevated DNA damage resulting in cancer cell death (Fig. 1 and Supplementary Fig. 1). Specifically, the reduced activation of Chek1/P53 cell cycle checkpoint in response to irradiation observed in AEP KD cells (Figs. 4B and C) explains why AEP deficiency in cancer cells resulted in genomic instability and cell death, due to their inability to properly activate Chek1/P53 cell cycle checkpoint in response to DNA damage.

Proper DNA damage repair requires the coordinated activation of ATR and the phosphorylation of H2AX to recruit the DNA repair machinery [48], but also the activation of cell cycle checkpoints to allow for DNA repair before the cell cycle can restart [51]. Furthermore, the inability to activate Chek1 in response to genotoxic stress and to induce cell cycle arrest to allow for DNA repair leads to aberrant mitosis, genomic instability, and cell death [52], features observed in AEP-deficient cancer cells (Fig. 1). In this context, we hypothesized that AEP deficiency in cancer cells would result in reduced levels of DNA repair, thus explaining the genomic instability and cell death observed. To evaluate this, we measured the efficiency of the homologous recombination and nonhomologous end joining pathways using the DR-GFP [53], SA-GFP [54] and EJ5-GFP [54] reporter assays (Supplementary Fig. 4A). Indeed, shRNA-mediated AEP KD drastically reduced homology-directed repair, both classical homologous recombination (as measured with the DR-GFP system; Fig. 4D, left panel) and single-strand annealing (as measured with the SA-GFP system; Fig. 4D, right panel), without affecting nonhomologous end-joining (as measured with the EJ5-GFP system; Fig. 4D, middle panel). Interestingly, homologous recombination contributes to genomic stability by facilitating DNA repair primarily during S and G2 phases [55], thus explaining the accumulation of DNA damage and increased genomic instability observed in AEP-deficient cancer cells. These data further support our previous observations and show that this new role of AEP is related to promoting genomic stability in BC cells and patients (Figs. 1 and 2L).

To further our understanding of the role of AEP in genotoxic stress tolerance in BC cells we aimed to investigate how AEP deficiency in BC cells resulted in reduced levels of both pChek1 and pP53, albeit increased levels of ATR. Our data, showing faster dephosphorylation of both pChek1 and pP53 in AEP-deficient cells, suggest that reduced phosphatase activity in BC cells could account for the prolonged activation of Chek1 and P53 shown in control MDA-MB-231 cells compared with AEP KD cells (Fig. 4C), with previous works suggesting a potential role for AEP as a regulator of protein phosphatase activity [56–58]. In this context, AEP deficiency in MDA-MB-231 cells resulted in increased protein phosphatase activity, as measured with 6,8-difluoro-4-methylumbelliferyl phosphate (DiFMUP), which could be dampened by pretreating AEP KD samples with recombinant AEP (Fig. 4E), supporting a role of AEP in DNA damage repair response and cell cycle regulation through the regulation of protein phosphatase activity in BC cells. Thus, we hypothesized that protein phosphatase inhibition prior to irradiation in AEP-deficient BC cells could rescue Chek1 and P53 phosphorylation. Interestingly, our proteomic data showed that AEP inhibition resulted in a twofold accumulation of PPP1R10 (*p* = 0.002) (Supplementary Table S1), a subunit of the PTW/PP1 complex involved in the regulation of the mitosis-to-interphase transition [59, 60]. It was previously known that genetic ablation of PPP1R10 results in prolonged Chek1 activation after ionizing-radiation-induced DNA damage [29], a phenotype that matches our observations in BC cells compared to AEP-deficient cells. This result revealed PPP1R10 as the potential AEP target responsible for the increased protein phosphatase activity and the reduced levels of pChek1 and pP53, and for the increased genomic instability observed in AEP KD MDA-MB-231 cells. To explore the link between AEP and the PTW/PP1 complex we decided to specifically inhibit PP1 and interrogate whether we could rescue Chek1 and P53 phosphorylation in AEP-deficient BC cells. Acute protein phosphatase inhibition using 1 μM okadaic acid, a PP1 specific inhibitor [61], 30 min prior to a single dose of irradiation (10 Gy) allowed for the recovery of both pChek1-S345 and pP53-S15 levels in AEP-deficient MDA-MB-231 cells (Fig. 4F), strengthening the idea that AEP functions as a regulator of pChek1 and pP53 through the regulation of protein phosphatase activity in cancer cells, through the regulation of PPP1R10 levels. Furthermore, a negative correlation between AEP and PPP1R10 at the protein level is also observed in BC patients (Figs. 3C and 4G), reinforcing a direct role for AEP in the regulation of PPP1R10 levels, and therefore in the regulation of protein phosphatase activity in BC. To validate the role of AEP in the regulation of PPP1R10 levels we checked whether AEP deficiency resulted in PPP1R10 accumulation. Indeed, AEP KD in MDA-MB-231 cells resulted in PPP1R10 accumulation at the protein level (Fig. 4H), without affecting its expression levels (Supplementary Fig. 4B), in agreement with a direct role of AEP in the regulation of PPP1R10 levels. Next, to explore whether the accumulation of PPP1R10 observed in AEP KD MDA-MB-231 cells results in increased levels of the PTW/PP1 complex we performed co-immunoprecipitation assays. Remarkably, even though AEK KD MDA-MB-231 cells showed greatly reduced levels of WDR82, one of the members of the PTW/PP1 complex [60], co-immunoprecipitation assays showed a three-fold increase in WDR82 in AEP KD MDA-MB-231 cells when PPP1R10 was immunoprecipitated compared to control cells (Supplementary Fig. 4C), further reinforcing a role for AEP in the regulation of PPP1R10 levels and PTW/PP1 complex. The direct role of AEP in the regulation of PPP1R10 levels was confirmed through PLA assays, which showed that a nuclear pool of AEP directly interacts with PPP1R10 within the nucleus of MDA-MB-231 cells (Fig. 4I). Finally, in vitro digestion assays demonstrated recombinant AEP is able to completely digest PPP1R10 at pH 7.2, further validating the direct role of AEP in the regulation of PPP1R10 levels (Supplementary Fig. 4D). These data reinforced a role for AEP in regulating the activity of protein phosphatases, allowing BC cells to uncouple DNA damage signaling and cell cycle checkpoint activation, and provide the molecular basis that allow to understand the genotoxic tolerance-mediated by AEP in cancer cells.

To further check the potential role of AEP in genotoxic stress tolerance in cancer cells, we decided to test whether AEP deficiency in MDA-MB-231 cells resulted in increased cell death in response to gamma-irradiation. Indeed, increased levels of cell death were observed in AEP-deficient cells compared to control cells after 48 h of gamma-irradiation (Supplementary Fig. 4E), reinforcing a novel role for AEP in genotoxic tolerance in BC cells. In addition, we overexpressed AEP in HCT116 cells, a cell model expressing low AEP levels (Supplementary Fig. 3A), and checked whether AEP overexpression reduced gamma irradiation-induced cell death. As shown in Supplementary Fig. 4F, AEP overexpression in HCT116 cells resulted in an increased cell death compared to control HCT116 even in the absence of irradiation. After 48 h irradiation, the percentage of cell death in control, 5 Gy treated cells increased up to 2.3-fold as compared to control, non-irradiated cells. However, no statistical differences were detected between irradiated vs non-irradiated cells in AEP overexpressing cells, supporting the role of AEP in radioresistance in cancer cells.

In conclusion, our data suggest that, in BC cells, AEP contributes to genotoxic tolerance by reducing DNA damage signaling through a reduction in ATR levels, while promoting sustained pChek1 and pP53 phosphorylation to arrest the cell cycle and promote DNA repair through the regulation of PPP1R10 levels and PTW/PP1 complex, thereby allowing cancer cells to scape DNA damage-induced cell death [62, 63].

### A high AEP-to-ATR protein ratio defines the response of patients with breast cancer to radiotherapy

**Fig. 5:**
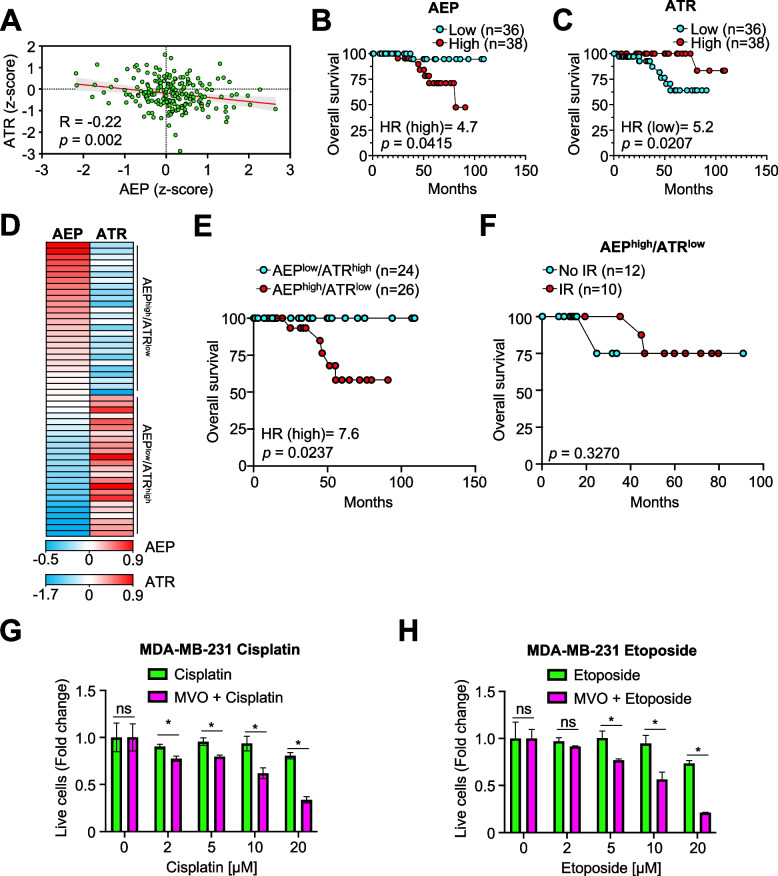
AEP contributes to breast cancer resistance to genotoxic stress. **A** Correlation analysis of AEP and ATR protein levels in BC patients using the TCGA breast carcinoma dataset. **B** Kaplan–Meier analysis in breast cancer patients expressing high (red dots) or low levels (cyan dots) of AEP at the protein level. **C** Kaplan–Meier analysis in BC patients expressing high (red dots) or low levels (cyan dots) of ATR at the protein level. **D** Heatmap showing the levels of protein (AEP and ATR, z-scores) expressed in BC patients. **E** Kaplan–Meier analysis in BC patients expressing AEP^low^/ATR^high^ levels (cyan dots) vs AEP^high^/ATR^low^ levels (red dots) at the protein level. **F** Kaplan–Meier analysis in BC patients showing AEP^high^/ATR.^low^ protein levels treated (red dots) or untreated (cyan dots) with radiation. **G** Dose response of cisplatin in MDA-MB-231 cells in the presence (magenta bars) or absence (green bars) of MVO. **H** Dose response of etoposide in MDA-MB-231 cells in the presence (magenta bars) or absence (green bars) of MVO. ns: no significant, * *p* < 0.05

Together, our data reveal a key role for AEP in BC resistance to genotoxic insults by proteolytically regulating ATR levels. Remarkably, despite the lack of correlation between AEP and ATR at the transcriptional level (Fig. 3C, G and H), a negative correlation in the protein levels of AEP vs ATR could be observed in TCGA BC patients (Figs. 3C and 5A), thus validating the findings in BC cells (Figs.3I and J). Interestingly, survival analyses using TCGA BRCA data demonstrated that patients with high AEP protein levels (AEP^high^, Fig. 5B) or low ATR protein levels (ATR^low^, Fig. 5C) had a worse prognosis and reduced overall survival, reinforcing their possible connection. However, to further investigate this novel AEP-ATR axis we analyzed whether AEP^high^/ATR^low^ patients presented differences in the overall survival compared with AEP^low^/ATR^high^ patients (Fig. 5D). These analyses revealed that AEP^high^/ATR^low^ patients had reduced overall survival, whereas AEP^low^/ATR^high^ patients had one hundred percent overall survival (Fig. 5E). Interestingly, AEP^low^/ATR^low^ or AEP^high^/ATR^high^ patients (Supplementary Fig. 5A) did not significantly differ in overall survival (Supplementary Fig. 5B), showing better overall survival than AEP^high^/ATR^low^ patients (Fig. 5E), suggesting that low ATR or high AEP levels alone are not sufficient to explain the reduced overall survival.

To explore the role of AEP in genotoxic tolerance in cancer, we investigated whether BC patients expressing different protein levels of AEP and ATR presented differences in the radiotherapy response. Patients with ATR^high^ levels had a good prognosis and no significant difference in overall survival, independent of whether they were treated or not with radiotherapy (Supplementary Fig. 5C). Next, we focused on those patients expressing ATR^low^ levels, who showed reduced overall survival (Fig. 5C). Remarkably, AEP^low^/ATR^low^ patients had a 100% percent overall survival rate when receiving radiotherapy, whereas untreated patients showed a reduced overall survival rate (Supplementary Fig. 5E). This finding further corroborates that ATR^low^ levels in BC patients are not sufficient to explain tumor resistance to genotoxic stress. On the other hand, patients with AEP^high^ levels showed worse prognosis (Fig. 5B), independent of whether they received radiotherapy or not (Supplementary Fig. 5D), thus supporting a direct role for AEP in cancer resistance to genotoxic insults. Interestingly, while AEP^high^/ATR^high^ patients exhibited a favorable overall survival rate (Supplementary Fig. 5B), the BC sample cohort with AEP^high^/ATR^low^ levels for whom data regarding radiotherapy treatment were available (*n* = 22), accounted for most of the reduced overall survival observed in AEP^high^ expressing patients (Fig. 5B), showing no significant difference independent of their being treated or not with radiotherapy (Fig. 5F). These data confirm the role of AEP in the regulation of tumor resistance to genotoxic stress through the regulation of ATR levels, with AEP^high^/ATR^low^ patients showing a limited response to radiotherapy. On the contrary, all the remaining patient groups had good overall survival and response to radiotherapy. Hence, our data reinforce a key role for this novel AEP-ATR axis in genotoxic tolerance in BC, suggesting the possibility of combining AEP inhibitors with current radiotherapy or chemotherapy approaches to increase treatment efficiency in AEP^high^/ATR^low^ patients.

Finally, to test our hypothesis, we studied whether AEP inhibition in BC cells sensitizes them to genotoxic insults. This study revealed that 24-h AEP inhibition in MDA-MB-231 cells sensitized them to the chemotherapeutic drugs cisplatin and etoposide, leading to increased cell death, further corroborating the role of AEP in BC cells resistance to genotoxic stress (Fig. 5G and H). Remarkably, similar findings were obtained in U2OS cells (Supplementary Figs. 5F and G), in which we previously showed that AEP-deficiency also resulted in cell death (Supplementary Fig. 1) and increased ATR levels (Supplementary Fig. 2), validating our hypothesis and the novel role of AEP as a regulator of ATR levels and as therapeutic target to sensitize cancer cells to genotoxic insults.

### Increased nuclear AEP levels reduce ATR nuclear accumulation in nonresponder invasive ductal breast carcinoma patients

**Fig. 6:**
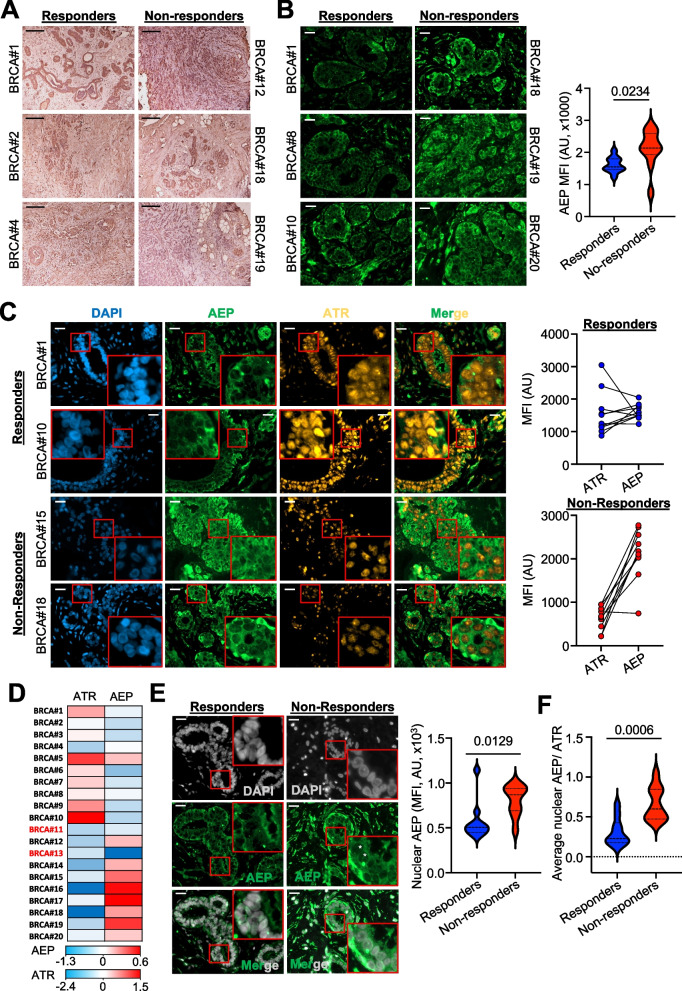
Increased AEP levels reduce ATR nuclear levels in ductal breast carcinoma cells in chemotherapy non-responder human patients. **A** Microscopy images (10x) of the immunohistochemical analysis of AEP expression in responder and non-responder invasive ductal breast carcinoma samples. (Size bar = 200 μm). **B** Immunofluorescence analysis of AEP expression levels in ductal breast carcinoma cells in responder and non-responder patients, alongside quantitation. Data represents the average AEP expression levels quantified using samples from 10 responder (50 cells per patient) and 10 non-responder (50 cells per patient) patients. (Size bar = 20 μm). **C** Immunofluorescence analysis of the expression levels of AEP and ATR in ductal breast carcinoma cells in responder and non-responder patients. Each pair represents the average ATR and AEP expression levels obtained upon quantification of data obtained per patient using 50 cells per patient. (Size bar = 20 μm). **D** Heatmap showing the protein levels (AEP and ATR, z-scores) expressed in invasive ductal breast cancer patients organized into responder and non-responder patients. **E** Immunofluorescence analysis of the nuclear AEP expression levels in ductal breast carcinoma cells in responder (*n* = 10) and non-responder (*n* = 10) patients, alongside quantitation. Data represents the average nuclear level of AEP obtained from the quantification of at least 80 cells per patient. (Size bar = 20 μm). White asterisk indicates AEP-positive nuclei in non-responder samples as compared to responder samples. **F** Nuclear AEP/ATR ratio obtained from responder (*n* = 10) and non-responder (*n* = 10) patients. Each dot represents the average nuclear AEP/ATR ratio obtained for each patient upon quantification of at least 80 cells per patient

Our previous data (Fig. 5 and Supplementary Fig. 5) suggest that increased AEP levels are linked to reduced radiotherapy response. Therefore, we propose that AEP could be a determinant of the level of radiotherapy resistance in BC patients. To validate whether AEP could discriminate the response to therapy, we analyzed the levels of AEP in a series of human IDBC patient samples previously classified as responders (*n* = 10) and nonresponder (*n* = 10). Immunohistochemical analyses revealed that AEP was expressed mainly in ductal BC cells (Fig. 6A and Supplementary Fig. 6A), with immunofluorescence approaches revealing higher AEP levels in ductal BC cells from nonresponder patients compared to responder patients (Fig. 6B). Next, we investigated whether a negative correlation between AEP and ATR levels could also be detected in these cancer cells. Indeed, higher AEP levels in nonresponder patients correlated with reduced ATR nuclear levels in 9 out of 10 patients (Fig. 6C), further confirming our previous observations revealing a direct role for AEP in regulating the levels of ATR and reinforcing their connection with tumor resistance to radiotherapy. Conversely, responder patients included those with AEP^low^/ATR^high^ levels or similar AEP-to-ATR levels (Fig. 6C), further reinforcing our previous observation that AEP^high^/ATR^low^ patients exhibited the poorest response to radiotherapy. Moreover, our analysis confirmed the AEP/ATR ratio as an efficient predictive biomarker for the stratification of responder (AEP^low^/ATR^high^, AEP^low^/ATR^low^ or AEP^high^/ATR^high^) and nonresponder (AEP^high^/ATR^low^) patients (Fig. 6D), further supporting an unexpected connection between AEP and ATR levels in genotoxic tolerance in cancer cells (Fig. 5). Moreover, our previous data indicate that AEP specifically accumulates in the nucleus of cancer cells; thus, we wondered whether AEP nuclear localization could also be observed in primary tumor samples supporting the idea that a nuclear pool of AEP specifically targets and fine-tunes ATR nuclear levels. Indeed, increased AEP nuclear levels were specifically detected in ductal BC cells from nonresponder patients (Fig. 6E). Furthermore, an increased nuclear AEP/ATR ratio (Fig. 6F and Supplementary Fig. 6B) was observed in nonresponder patients. In this context, on average, 60% of nonresponder patients had ductal carcinoma cells with a nuclear AEP/ATR ratio greater than 0.5, whereas 16% of responder patients did. These combined data stress the clinical relevance of the direct role of AEP in the fine-tuning of ATR levels in BC cells and in their resistance to radiotherapy.

## Discussion

Understanding tumor resistance and identifying novel druggable targets to sensitize them to radio- or chemotherapy approaches is a major research focus in oncology. This is because, despite current advancements in both strategies that have contributed to improve treatment outcomes [1], cancer resistance still accounts for 80–90% of deaths in cancer patients [2, 3].

AEP overexpression correlates with poor prognosis, increased malignancy, and worse overall survival in many human solid tumors [16, 19, 20, 22, 23, 25, 31, 64]. Remarkably, novel roles of AEP in tumor onset and progression are emerging, with the identification of the molecular targets of AEP allowing to understand its role in cancer biology. Recent work demonstrates that AEP suppresses lung metastasis, suggesting a potential role for AEP in BC onset and progression [17]. Furthermore, AEP promotes glioblastoma tumorigenesis and proliferation through p53 inactivation [16], facilitates tumor malignancy through TMOD3 processing [30], and mediates cancer cell adaptation to harsh environments through DDX3X cleavage [18]. Together, these studies strongly support a key role for AEP in cancer, highlighting the importance of identifying novel targets and biological processes regulated by AEP contributing to the onset and progression of this disease. Here, we reveal AEP as a determinant of radiotherapy resistance in BC. Our data show that AEP accumulates in radiotherapy nonresponder invasive breast carcinoma patients, playing a dual role in tumor resistance to genotoxic stress. Moreover, we reveal AEP as a previously unidentified predictive biomarker for radioresistant BC patients and as a therapeutic target for sensitizing BC cells to current radiotherapy and chemotherapy approaches.

At the mechanistic level, we demonstrate that AEP functions as a determinant of genotoxic stress tolerance in BC by uncoupling DNA damage signaling from cell cycle checkpoints and DNA damage repair. First, our work shows that AEP suppresses ATR levels, thus reducing DNA damage-induced apoptosis in response to genotoxic stress [65–68]. Importantly, reduced ATR levels in BC patients have been shown to correlate with reduced overall survival [69]. Furthermore, ATR haploinsufficiency resulting in reduced ATR activity has been revealed as a driver of tumorigenesis [70–74]. In this context, reduced ATR levels are tumor-prone due to partial DNA damage response defects leading to increased genomic instability [72]. However, the mechanisms controlling ATR levels remain unclear. Our work directly elucidates AEP as a previously unidentified regulator of ATR levels in cancer, thus allowing cancer cells to better cope with genotoxic stress. Notably, our proteomics data indicate ATR is not the only protein involved in DDR and genomic stability regulated by AEP, suggesting AEP plays a broader role in the regulation of these key biological processes, that will require further investigation.On the other hand, we show that AEP reduces PPP1R10 levels, leading to sustained Chek1 and P53 phosphorylation, thus promoting the activation of the G2/M checkpoint and facilitating DNA damage repair in response to genotoxic insults in BC. Interestingly, genetic ablation of PPP1R10, a regulatory subunit of PP1 [59], results in prolonged Chek1 and P53 activation after ionizing-radiation-induced DNA damage [29], a phenotype that matches our observations in BC cells compared to AEP-deficient cells. Furthermore, reduced PP1 levels have been shown to promote tumorigenesis and to correlate with reduced overall survival [75–78], however the underlying mechanisms controlling PP1 activity in BC cells remain unclear. Our study reveals AEP as a regulator of cell cycle progression in response to genotoxic stress through the modulation of PPP1R10 levels, thus explaining the reduced levels of pChek1 and pP53, reduced DNA damage repair, and increased genomic instability observed in AEP KD MDA-MB-231 cells. Taken together, our findings provide the underlying mechanisms to understand the role that AEP plays in tumor resistance against genotoxic stress.

The clinical significance of our findings is demonstrated by our analyses revealing that radioresistant BC patients are efficiently identified as AEP^high^/ATR^low^ BC patients. First, we reveal that AEP accumulates in ductal breast carcinoma cells from nonresponder patients, resulting in reduced ATR nuclear levels, with these patients showing a higher AEP/ATR ratio. Conversely, AEP^low^/ATR^high^, AEP^low^/ATR^low^ or AEP^high^/ATR^high^ patients are efficiently discriminated as responders. Thus, our work identifies for the first time the AEP/ATR axis as a predictive biomarker for the identification of radiotherapy nonresponder patients. Moreover, we demonstrate that AEP inhibition in BC cells sensitizes them to chemotherapeutic reagents such as cisplatin or etoposide, revealing AEP as a promising therapeutic target for the treatment of radioresistant BC patients. Importantly, our data reveal a similar relationship between AEP and ATR levels not only in BC, but also in other types of cancer. Specifically, our findings indicate that in cancers where high AEP expression levels are associated with poor prognosis, the opposite is observed for ATR. Thus, these observations suggest a more general role for this newly identified AEP/ATR axis in cancer, although the connection between the AEP/ATR axis and tumor resistance in other types of cancer remains to be explored. The present study reveals the AEP/ATR/PPP1R10 axis as a determinant of genotoxic stress tolerance and radiotherapy resistance in BC and provides the theoretical basis for the design of novel AEP-targeted therapies in the treatment of chemotherapy or radiotherapy resistant BC tumors.

## Conclusion

**Fig. 7:**
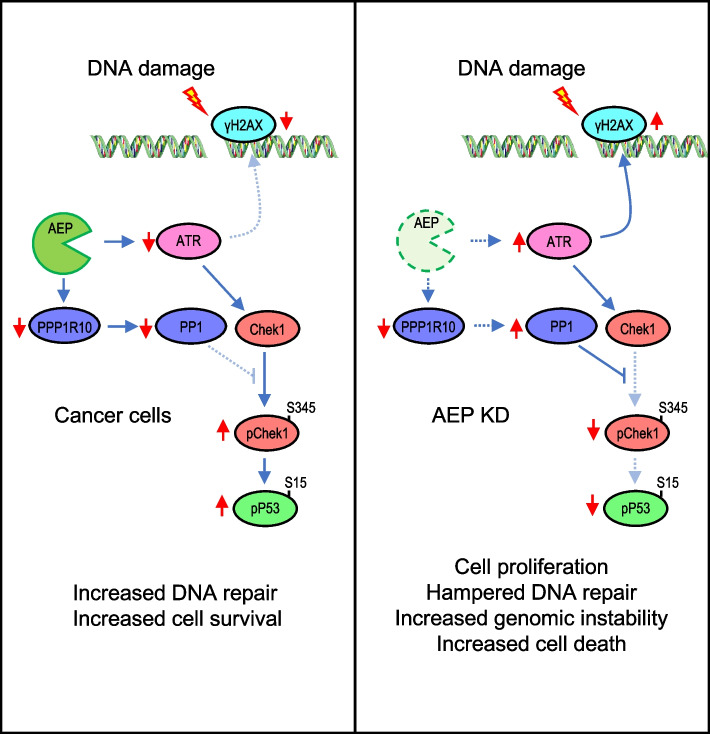
AEP plays a dual role in radiotherapy resistance in BC patients. In BC patients, AEP reduces DNA damage response by suppressing ATR levels, while maintaining sustained Chek1 and P53 activation through the suppression of PPP1R10 levels and PP1 activity, thus allowing cancer cells to scape DNA damage-induced cell death and to efficiently repair DNA damage, thus explaining the role of AEP in radiotherapy resistance (left panel). Conversely, AEP deficiency in cancer cells result in increased ATR levels leading to elevated DNA damage signaling and increased PP1 activity, resulting in reduced levels pf pChek1 and pP53 leading to reduced DNA repair, increased genomic instability and cell death (right panel)

In the current study we identify the role of the AEP/ATR/PPP1R10 axis in genotoxic stress tolerance in breast cancer patients. We demonstrate that AEP suppresses ATR levels resulting in reduced levels of DNA damage signaling, thus allowing BC cells to scape DNA damage-induced cell death. Besides, we reveal AEP reduces protein phosphatase activity through the suppression of PPP1R10 levels in BC, thus allowing cell cycle checkpoint activation and efficient DNA damage repair. Therefore, our work uncovers a dual role for AEP in genotoxic stress resistance in BC by uncoupling DNA damage response from cell cycle checkpoint activation (Fig. 7). Furthermore, we provide strong evidence supporting AEP as both, a novel therapeutic target and an efficient predictive biomarker for the treatment and stratification of nonresponder patients.

## Materials and methods

### Human tumor samples

A total of 20 paraffin-embedded human breast cancer (BC) samples at diagnosis were provided by the Biobank of the Anatomy Pathology Department (record number B.0000745, ISCIII National Biobank network) of the MD Anderson Cancer Centre Madrid, Spain. In summary, the patient samples were selected according to pathological criteria as infiltrating ductal breast carcinoma diagnosed as G2-3. Patients, aged 49 + 8 years without previous oncological diseases, received standard breast cancer treatment and radiotherapy as appropriate [79]. The response to radiotherapy was assessed according to established criteria [80]. This study was conducted in accordance with the ethical standards set forth in the Spanish legislation (Ley de Investigacion Organica Biomedica, 14 July 2007) and was approved by the ethical committees of the MD Anderson Cancer Centre Madrid, Spain. A comprehensive written informed consent was obtained from all participants.

### Cell culture

Cell lines were acquired from the American Type Culture Collection (ATCC). HEK293 cells (RRID:CVCL_0045) were cultivated in low-glucose DMEM (Sigma, #5546), supplemented with 100U/ml penicillin, 100 μg/ml streptomycin and 2 mM L-glutamine. Cancer cell lines (*i.e.,* HCT116 (RRID:CVCL_0291), MDA-MB-231 (RRID:CVCL_0062), U2OS (RRID:CVCL_0042), U87 (RRID:CVCL_0022) and T98 (RRID:CVCL_B368)) were cultured in high-glucose DMEM containing 100U/ml penicillin, 100 μg/ml streptomycin, 2 mM L-glutamine. All cell lines were cultured at 37ºC in an atmosphere of 5% CO_2_.

### Antibodies

All antibodies used in this manuscript were used a 1:1000 dilutions for immunoblotting or 1:200 dilutions for immunofluorescence. Antibodies against GAPDH (clone 14C10, #2118), COXIV (clone 3E11, #4850), β-actin (clone 13E5, #4970), P53 (clone 1C12, #2524), pS15-P53 (#9284), pS345-Chek1 (clone 133D3, #2348) and anti-cleaved caspase 3 (clone 5A1E, #9664) were purchased from Cell Signaling. Antibodies against Lamp2 (clone H4B4, #ab25631), CtsD (clone EPR3057Y, #ab75852) and Glb1 (EPR8250, #ab128993) were obtained from abCam. Antibodies against H3 (#SAB4200651) and pS139-H2AX (clone JBW301, #05–636) were acquired from Sigma. Antibodies against GFP (#A-11122), KU80 (clone 9403, #MA1-23,314) and ATR (#PA5-85,507) were obtained from Thermo Scientific. Antibody against MRE11A (#203,501-T38) was acquired from SinoBiologicals. Antibodies against CHEK1 (clone G4, #sc-8408) and TOP2A (clone F12, #sc-365916) were obtained from SantaCruz. Antibodies against PPP1R10 (#24,450.1.AP) and WDR82 (#21,354.1.AP) were purchased from ProteinTech. Horseradish peroxidase-conjugated streptavidin (SAV-HRP, #016–030–084) was obtained from Jackson Immunoresearch. Sheep anti-AEP was obtained as previously described [81]. Briefly, sheep were immunized with a mixture of the 56 kDa precursor and 46 kDa AEP and affinity purified on immobilized 56 kDa full length AEP, obtaining a polyclonal antibody able to recognize both the 56 kDa precursor and the mature forms of AEP.

### Sample preparation and immunoblotting

Cell samples for immunoblotting were prepared in complete RIPA buffer supplemented with protease inhibitor cocktail (Roche, 11,836,170,001) and MVO 10 μM. For cell signaling immunoblots to maintain endogenous phosphorylation levels phosphatase inhibitor cocktails 2 and 3 (Sigma, P5726 and P0044) were added to the lysis buffer (RIPA buffer supplemented with protease inhibitor cocktail (Roche, 11,836,170,001) and MVO 10 μM).

### PP1 inhibition

Both, control and AEP KD MDA-MB-231 cells were pre-treated with 1 μM okadaic acid for 30 min prior to a single dose of irradiation (10 Gy). After 2 h recovery, cells were lysed in complete RIPA buffer supplemented with protease inhibitor cocktail (Roche, 11,836,170,001), MVO 10 μM and phosphatase inhibitor cocktails 2 and 3 (Sigma, P5726 and P0044) and used for immunoblotting assays.

### Co-immunoprecipitation assay

Control and AEP KD cells were washed twice with cold PBS and lysed in 300 µl of IP lysis buffer (20 mM Tris pH 7.5, 150 mM NaCl, 1 mM EDTA, 1% NP40), supplemented with protease inhibitor cocktail P8340 (Sigma), 1 mM PMSF, 10 µM E64d and 1 µM pepstatin. After centrifugation (21,000 × g 4ºC, 5 min), 50 µl was used as whole-cell extract (WCE) and 250 µl was used for the Co-IP protocol. Cell lysates were incubated with Dynabeads Protein G (Thermo Fisher Scientific, 10004D) coupled to the appropriate antibody for 45 min at RT according to the manufacturer’s protocol. The immunoprecipitated magnetic beads were then washed 3 times with IP lysis buffer and the bound proteins were eluted using a standard denaturing elution protocol. Briefly, the immunoprecipitated beads were resuspended in 50 µl of elution buffer (pre-mixed IP lysis buffer and Laemmli buffer with 2-mercaptoethanol). WCE and immunoprecipitated proteins were used for immunoblotting analysis.

### Tissue immunohistochemistry and immunofluorescence in human samples

Paraffin-embedded samples were submitted to standard immunohistochemistry (IHC) protocol. Briefly, after deparaffinization and hydration, antigen retrieval was performed with 10 mM citrate buffer pH 6.0 in microwave, followed by peroxidase inhibition with 3% H_2_O_2_ and blocking of unspecific binding with 5% BSA. Incubation with primary antibody anti- AEP 1:100 at 4 °C overnight followed by secondary anti-sheep-HRP for 1 h at RT. DAB reaction kit (catalogue no. SK-4100) was used to visualize the peroxidase reaction product. Samples without the primary antibody were used as a negative control. All the sections were counterstained with hematoxylin. Images were obtained with a Leica microscope and Leica digital camera. For immunofluorescence staining the peroxidase inhibition step was omitted. After blocking, sections were incubated with anti-AEP (as mentioned above) and anti-ATR 1:200 at 4 °C overnight, followed by anti-sheep and anti-mouse Alexa Fluor-conjugated secondary antibodies, respectively. Sections analyzed and images acquired with a Zeiss ApoTome 3 microscope.

### Enzymatic activity assays

Total cell lysates were prepared in 0.2 M Na citrate buffer pH 4.0 containing 1% Triton X-100 (Sigma, #T9284). AEP activity was determined using 20 μg of total protein in 200 μl of assay buffer (0.2 M Na citrate buffer pH 4.0, supplemented with 1 mM DTT) containing 10 μM of AEP substrate (Z-Ala-Ala-Asn-7-amino-4-methylcoumarie (AMC), (Bachem, #4,033,201)). Release of AMC was quantified at 460 nm in a fluorescence plate reader (Fluorostar Optima, BMG Labtech).

### In vitro digestion assay

Recombinantly expressed proteins were extracted in 50 mM sodium acetate buffer containing 1% Triton X-100, containing 4 mM EDTA, pH7.2 and supplemented with protease inhibitor cocktail (Roche, 11,836,170,001). Equal amounts of protein (20 μg) were incubated in a pH-modified AEP reaction buffer (50 mM sodium acetate, 4 mM EDTA, pH7.2 to mimic nuclear pH as previously reported [41–45], 5 mM DTT and protease inhibitor cocktail) for 3 h at 37ºC in the presence or absence of recombinant AEP. When indicated, 1 μM MVO was added to recombinant AEP for 30 min before the in vitro digestion reaction to block AEP.

#### Phosphatase activity assay

Total cell lysates from control and AEP deficient MDA-MB-231 cells were prepared in Tris–HCl 50 mM, pH 8 buffer, containing 100 mM NaCl, 2 mM EDTA and 1% NP-40, and supplemented with Protease Inhibitor Cocktail and PMSF. All samples were incubated at 37ºC for 30 min in the presence or absence of recombinantly expressed AEP, prior to the measurement of phosphatase activity. Phosphatase activity was measured using DiFMUP (6,8’-Difluoro-4-Mrthylumbelliferyl Phosphate) following manufacturer instructions.

#### Immunofluorescence microscopy

Cells (5 × 104 cells/coverslip) were fixed in PBS containing 4% paraformaldehyde for 20 min at room temperature. Then, cells were washed with PBS three times, permeabilized with 0.2% Triton X-100 in PBS for 5 min at room temperature, washed again three times with PBS and incubated in blocking solution (1% BSA in PBS) for 20 min at room temperature. Then, cells were stained using a 1:200 dilution of primary antibody in blocking solution for 45 min at room temperature, washed three times with PBS and stained with a 1:500 dilution of the Alexa-488 or Alexa-594 conjugated secondary antibody (Invitrogen). For subcellular localization experiments, cells were stained with anti-AEP or a sheep IgG antibody (Thermo, #31,243) as negative control (1:200). Finally, coverslips were washed three times in PBS and mounted using Prolong Gold Antifade with DAPI (Thermo Scientific) and images acquired using a LSM700 confocal microscope (Leica) or a Zeiss Apotome 3. Quantitative analyzed of the immunofluorescence images were done using ImageJ (RRID:SCR_003070) software.

#### Proximity ligation assay

Both control and AEP KD MDA-MB-231 cells were seeded on coverslips and grown for 24 h at 37ºC and 5% CO_2_. Then, cells were fixed in PBS containing 4% paraformaldehyde for 20 min at room temperature, permeabilized with ice-cold methanol for 20 min at −20ºC and stained with anti-AEP and anti-ATR or anti-AEP and anti-PPP1R10 (1:200) for Proximity Ligation Assays following manufacturer instructions (Sigma).

#### DNA constructs

DNA sequences coding for Nucleolar Protein 56 (NOP56, #HG16916-U), Nucleolar Protein 58 (NOP58, #HG20632-U), DNA replication licensing factor MCM6 (MCM6, #HG14317-G), DNA replication licensing factor MCM7 (MCM7, #HG16465-G), Heat shock protein HSP 90 alpha (HSP90AA1, #HG11445-G) and Heat shock protein HSP 90 beta (HSP90AB1, #HG11381-G) were obtained from SinoBiological, cloned into pEGFP-N1 (Clontech, #6085–1) and transformed into HEK293 (RRID:CVCL_0045) cells using JetPEI (Polyplus, #101,000,053) following manufacturer protocol. DNA coding for AEP was cloned into pSEMS-meGFP [82].

#### Sample preparation for label-free mass spectrometry

HEK293 cells were treated or not with 50 μM MVO26630 for 24 h. Next, cells were washed twice with ice-cold PBS, scrapped from cell culture dishes and collected by centrifugation at 300 g for 5 min at + 4ºC, and resuspended in SDS-containing lysis buffer (1% SDS in 100 mM Triethylammonium Bicarbonate buffer (TEAB, Sigma, #T7408 supplemented with protease inhibitor cocktail (ROCHE, 11,836,170,001) and 50 μM MVO)), kept on ice for 10 min to guarantee cell lysis. Then, cell lysates were centrifuged at 20,000 g for 10 min at + 4ºC and the supernatant transferred to a clean low-protein binding tube. Protein concentration was determined using the BCA Protein Assay Kit (Thermo Scientific, #23,227), and 1 mg of protein per experiment was reduced with 10 mM DTT (Sigma, #D0632) for 1 h at 55ºC and then alkylated with 20 mM iodoacetamide (IAA, Sigma, #6125) for 30 min at RT. Protein was then precipitated overnight at −20ºC using six volumes of chilled (−20ºC) acetone. After precipitation, protein pellet was resuspended in 1 ml of 100 mM TEAB and digested with Trypsin (1/100 w/w, Thermo, #90,058) and digested overnight. Finally, digested samples were cleared by centrifugation at 20,000 g for 30 min at + 4ºC, and peptide concentration quantified with a Quantitative Colorimetric Peptide Assay Kit (Thermo, #23,275).

#### Mass spectrometry

Peptide samples were analyzed using a nanoflow liquid chromatography system (Ultimate 3000 RSLCnano system, Thermo Scientific) coupled to an LTQ Orbitrap Velos Pro mass spectrometer (Thermo Scientific). Samples, usually 10 μl, were loaded onto a C18 trap column and washed for 5 min with 0.1% formic acid. The peptides were resolved using a gradient (130 min, 0.3 μl/min) of buffer A (0.1% formic acid) and buffer B (80% acetonitrile in 0.08% formic acid): 2% buffer B for 4 min, 2%−40% buffer B for 64 min, 40%−98% buffer B for 2 min, 98% buffer B for 15 min, 98%−2% buffer B for 1 min and 2% buffer B for 44 min. Peptides, initially trapped on an Acclaim PepMap 100 C18 column (100 μm x 2 cm, Thermo Scientific), were then separated on an Easy-Spray PepMap RSLC C18 column (75 μm x 50 cm, Thermo Scientific), and finally transferred to a LTQ Orbitrap Velos Pro mass spectrometer via an Easy-Spray source set at 50ºC and a source voltage of 1.9 kV. For the identification of the peptides, a top 15 method (1 MS plus 15 MS^2^, 100 min acquisition) consisting of full scans and mass range (m/z) between 335 and 1,800 (m/z). The Orbitrap was operated in a profile mode, resolution of 60,000 with a lock mass set at 445.120024 and a max fill time of 500 ms. LTQ was operated in a centroid mode with isolation width = 2.00 (m/z), normalized collision energy = 35.0, activation time = 10 ms and max fill time of 100 ms.

#### Mass spectrometry analyses

LTQ Orbitrap Velos Pro.RAW files were analyzed, and peptides and proteins quantified using MaxQuant [83]. All settings were set as default, except for the minimal peptide length of 5, trypsin was specified as the proteolytic enzyme with up to 2 miss cleavages allowed, and Andromeda search engine was configured for the Uniprot Homo sapiens protein database. Peptide and protein ratios only quantified in at least two out of the three independent, biological replicas were considered. Statistical significance was calculated using Student’s t test.

#### Gene ontology analyses

Gene Ontology (GO) analyses to reveal statistically over-represented terms were performed using ClueGo [84]. The proteins accumulating upon AEP inhibition or biotinylated when using AEP-TiD in our mass spectrometry analyses were used as a test dataset and a reference set of GO annotations for the human proteome was used to carry out the GO enrichment analyses. Analyses were done using a right-sided hypergeometric test and only GO terms (*i.e.,* cellular location and biological process) with *p* < 0.005 were selected.

#### Subcellular fractionation

HEK293, HCT116, MDA-MB-231, U2OS, T98 and U87 cells cultured in 6-well plates were lysed using ProteoExtract Subcellular Proteome Extraction Kit (Sigma, #539,790) following manufacturer instructions to obtain cytoplasmic, membrane and nuclear fractions, and used for immunoblotting analysis of the subcellular localization of AEP in the nuclear and membrane fractions, and also to analyze enzymatic activity of AEP in the nuclear fraction, as previously described [34].

#### Cell proliferation analyses

HCT116, MDA-MB-231or U2OS (3 × 10^4^ cells) were cultured in 6-well plates and cultured for 24, 48 and 72 h in the presence or absence of 50 μM MVO. At the times indicated cells were detached by trypsinization, washed twice with ice-cold PBS, resuspended in 1 ml of DMEM and counted using a hemocytometer. Each data point was counted at least 5–6 time, using their average to increase the accuracy of the data. Data represents the mean ± SEM from at least 5 independent, biological replicas.

#### Generation of stable AEP knock-down cancer cell lines by lentiviral infection

To generate stable AEP knock-down cancer cell lines (MDA-MB-231 and U2OS), MISSION shRNA against AEP (Sigma, #SHC001) in pLKO.1-Puro (RRID:Addgene_139470) was purchased from Sigma (5’- GTATTGAGAAGGGTCATATTT-3’, #TCRN0000276301). Lentivirus were produced upon transfection of HEK293 cells using the calcium phosphate protocol using psPAX2 (lentiviral packaging plasmid) and pMD2.G (envelope expressing plasmid) (RRID:Addgene_12259) vectors. Supernatant containing the lentivirus were collected 48 h after transfection and concentrated by ultracentrifugation at 22,000 rpm for 90 min at + 4ºC (Beckman Coulter Optima TM L-100 K). MDA-MB-231 and U2OS were plated (6 × 10^5^ cells) in 10 cm dishes and transduced with lentiviral particles carrying shRNA against AEP or scramble shRNA (5’-CTTTGGGTGATCTACGTTA-3’) 2 days later. Stable populations of stable AEP knock-down cell lines for both MDA-MB-231 and U2OS cells were obtained after selection of infected cells in high-glucose DMEM containing 1 μg/ml puromycin (Gibco, #A1113803).

#### Cell cycle analyses

Stable AEP knock-down MDA-MB-231 and U2OS cells were trypsinized, washed three times using ice-cold PBS, and 2 × 10^6^ cells were fixed using 70% ethanol at −20ºC overnight. Then, cells were collected by centrifugation at 200 g for 10 min at + 4ºC, washed carefully three times with ice-cold PBS and then stained in 500 μl of propidium iodide (PI) staining solution (0.1% Triton X-100, 20 μg/ml PI (Sigma, #P4864) and 100 μg/ml DNAse-free RNAse A (Sigma, #10,109,142,001) in PBS) with incubation at 37ºC for 15 min in the dark. Samples were analyzed in a LSRFortessa X-20 (BD Biosciences) and the quantitative analyses were carried out using FlowJo software (Becton Dickinson, v.10.7.2) (RRID:SCR_008520).

#### Cell viability analysis

To assess cell proliferation, viability, and death, cells were seeded in 125.000 cells per mL in 24-well plates counting in triplicate. The number of totals, alive cells and dead cells was determined using trypan blue 5% solution (Bio- Rad) in triplicates and by two independent researchers.

#### DR-GFP, SA-GFP and EJ5-GFP assays

U2OS cells stably carrying a single copy of the DR-GFP, SA-GFP or EJ5-GFP reporters were used to analyze the role of AEP in the different double-strand break repair pathways, as previously described [85]. Cells were seeded in 6-well plates and infected with lentiviral particles containing the AEP shRNA-pLKO.1 construct using 8 μg/mL polybrene. The next day cells were infected with lentiviral particles harboring the I-SceI-BFP expression construct. Forty-eight hours later, cells were collected by trypsinization, fixed with 4% paraformaldehyde for 20 min and analyzed with a BD FACS LSRFortessa X-20 and the FACS DIVA software. The number of GFP-positive cells from at least 10,000 events positive for blue fluorescence (infected with the I-SceI-BFP construct) was scored. The data represents the mean and SD from at least three independent experiments.

#### Kaplan–Meier survival curves and AEP expression levels in cancer

The expression levels of AEP in human solid tumors vs normal tissue samples were analyzed using the GEPIA2 online tool and The Cancer Genome Atlas (TCGA) database. Kaplan–Meier survival analyses and gene expression box plots were calculated from the GEPIA2 online tool [32]. Kaplan–Meier survival analyses were performed using the GEPIA2 online tool to interrogate the effect of changes in the expression levels of AEP in the overall survival and prognosis in different human solid tumors.

#### Breast cancer patient data

Data from breast cancer patients was obtained from the TCGA database through the cBioportal website. Kaplan–Meier analyses and correlation analyses were done using GraphPad Prism software 9 (GraphPad Software, San Diego, CA, USA) (RRID:SCR_002798).

#### Data representation and statistics

All data analyses, representations and statistical analyses in this manuscript were performed using GraphPad 9 (GraphPad Software, San Diego, CA, USA). Statistical significance was calculated using unpaired Student´s t-test, unless otherwise stated, using GraphPad 9.

## Supplementary Information

Additional file 1. Supplementary Figure 1. AEP deficiency in cancer cells leads to cell cycle arrest and cell death. (A) Immunoblot of AEP in different cancer cell lines untreated or treated with 50 μM MVO for 24 h, alongside quantitation. (B) Immunoblot showing the shRNA-mediated AEP knock-down in U2OS cells. (C) Cell cycle analyses of control and AEP shRNA transduced U2OS cells. Data represents average ± SD of 4 independent, biological replicas. * *p* value <0.01. (D) Representative image of the cell cycle profile of control (upper panel) compared to AEP KD (lower panel) MDA-MB-231 cells indicating the percentage of cells in the different phases of the cell cycle. (E) Representative image of the cell cycle profile of control (upper panel) compared to AEP KD (lower panel) U2OS cells indicating the percentage of cells in the different phases of the cell cycle. (F) Micrographs showing the presence of micronuclei in U2OS cells upon shRNA-mediated AEP KD alongside quantitation. Quantitation represents the average ± SD of three independent experiments, each one including more than 100 cell. Size bar = 27um. (G) Micrographs showing examples of polyploid cells in shRNA-mediated AEP KD U2OS cells. (H) Micrographs showing internuclear DNA bridges in shRNA-mediated AEP KD U2OS cells. Arrowheads indicate DNA bridges. Size bar = 27um. (I) Micrographs showing γH2AX-positive internuclear DNA bridges in shRNA-mediated AEP KD MDA-MD-231 cells. (J) Micrographs showing anti-γH2AX staining in both control (left panels) and AEP shRNA transduced (right panels) U2OS cells alongside quantitation representing the median. (n>400 cells). Size bar = 27um.

Additional file 2. Supplementary Figure 2. Overlap and correlation of the intensities of the peptides identified in the individual biological replicas. (A) AEP activity in MEFs, HEK293 and HeLa cells. (B) Inhibition of AEP activity in HEK293T using different concentrations of MVO26630 (0, 2, 10 or 50μM) for 16 hours. Data represents the mean ± SD of three independent, biological replicas. (C) Proteomics workflow. (D) Percentage of shared peptides identified in the individual biological replicas. (E) Correlation of the intensities of the peptides identified in the individual biological replicas. (F) AEP activity measured in nuclear extracts of HEK293 cells and the effect of the specific AEP inhibitor (MVO26630). (G) Examples of Asn/Asp containing peptides accumulating upon MVO-mediated AEP inhibition highlighting the newly identified putative cleavage sites (in green). (H) In vitro digestion of the GFP-tagged, AEP putative targets using recombinant AEP at pH7.2 for 3 hours, alongside COXIV as negative control.

Additional file 3. Supplementary Figure 3. AEP regulates the levels of some of the identified targets in human cancer cells. (A) Immunofluorescence showing the subcellular localization of AEP in HCT116 (n>100 cells), U2OS (n>160 cells), U87 (n>120 cells) and T98 (n>90 cells), alongside quantitation showing for each cell line the cytoplasmic (C) and nuclear (N) intensity, corrected using a sheep IgG antibody. (Size bar=6um). (B) Immunoblot showing the nuclear (N) and membrane/organelle (M/O) localization of AEP in HCT116, U2OS, U87 and T98 cells, including Lamp2 as a lysosomal membrane marker, Glb1 and CtsD as soluble lysosomal hydrolases and H3 as a nuclear marker. (C) Correlation analysis of the protein expression levels of some of the novel AEP targets identified in our proteomic analysis vs AEP in breast cancer patients using data obtained from the TCGA database. (D) Kaplan-Meier analyses of patients of different types of cancer (Colon adenocarcinoma with high microsatellite instability (COAD MSI-H), Glioblastoma (GBM), Head and Neck Squamous cell Carcinoma (HNSC), Uveal Melanoma (UVM), Pheochromocytoma and Paranglioma with Wnt-altered (PCPG), Stomach Adenocarcinoma (STAD) and Rectum Adenocarcinoma (READ)) expressing high (red line) or low (blue line) levels of AEP (upper panels) or ATR (lower panels). (E) Immunoblot showing the effect of MVO-mediated AEP inhibition in the levels of ATR in U2OS cells alongside quantitation. Data represents the average ± SD of three independent, biological replicas. (F) Proximity ligation assay (PLA) using anti-AEP and anti-ATR in control and AEP KD MDA-MB-231 cells.

Additional file 4. Supplementary Figure 4. Role of AEP in genotoxic tolerance in BC cells. (A) Schematic representation of the systems used to measure the efficiency of DNA repair by different DNA repair mechanisms: classical homologous recombination (DR-GFP), single-strand annealing (SA-GFP) and non-homologous end-joining (EJ5-GFP). (B) qPCR analyses of the mRNA expression levels of AEP and PPP1R10 in control (EV, empty vector) and AEP shRNA (left panel) or control (NT, non-targeting) and AEP siRNA KD (right panel) MDA-MB-231 cells. (C) Co-immunoprecipitation assay of WDR82 using anti-PPP1R10 in control and AEP KD MDA-MB-231 cells. In red box cropped, overexposed WDR82 immunoblot. (D) In vitro digestion of PPP1R10 using recombinant AEP at pH7.2 for 3 hours. (E) Cell death in control and AEP KD MDA-MB-231 after 48 hours irradiation using 0, 2, 5 or 10 Gy. (F) Cell death in non-transfected vs AEP overexpressing HCT116 cells after 24- or 48-hours irradiation with 0 or 5 Gy. * *p*<0.05, ns: no significant.

Additional file 5. Supplementary Figure 5. AEP inhibition sensitizes cancer cells to genotoxic stress. (A) Heatmap showing the levels of protein (AEP and ATR) expressed in breast cancer patients dividing patients in four groups (AEPhigh/ATRlow; AEPlow/ATRhigh; AEPlow/ATRlow; AEPhigh/ ATRhigh). (B) Kaplan-Meier analysis in AEPlow/ATRlow (cyan dots) or AEPhigh/ATRhigh (red dots) breast cancer patients at the protein level. (C) Kaplan-Meier analysis in breast cancer patients expressing high ATR protein levels treated (red dots) or untreated (cyan dots) with radiation. (D) Kaplan-Meier analysis in breast cancer patients expressing high protein AEP levels treated (red dots) or untreated (cyan dots) with radiation. (E) Kaplan-Meier analysis in AEPlow/ATRlow breast cancer patients treated (red dots) or untreated (cyan dots) with radiation. (F) Dose response of cisplatin in U2OS cell in the presence (magenta bars) or absence of MVO (green bars). (G) Dose response of etoposide in U2OS cell in the presence (magenta bars) or absence of MVO (green bars). ns: no significant, * *p* < 0.05.

Additional file 6. Supplementary Figure 6. Increased AEP/ATR levels in ductal invasive breast carcinoma non-responder patients. (A) Microscopy images (40x) of the immunohistochemical analysis of AEP expression in responder and non-responder invasive ductal breast carcinoma samples (Size bar = 40μm). (B) Nuclear AEP/ATR ratio obtained from responder (*n*=10, blue dots) and non-responder (*n*=10, red dots) invasive ductal breast carcinoma patients. Each dot represents the nuclear AEP/ATR ratio obtained for each ductal breast carcinoma cell (n>80 cells per patient). Two-way ANOVA was used to calculate the statistical significance between responder and non-responder groups. Red dotted line indicates a nuclear AEP/ATR ratio greater than 0.5.

Additional file 7. Supplementary Table S1. HEK293 vs MVO Proteomics dataset – Protein groups

Additional file 8. Supplementary Table S2. HEK293 vs MVP Proteomics dataset – Peptide Cleavage Sites

## Data Availability

All data generated in this work is available from the authors upon request. Proteomics data generated in this study are publicly available in ProteomeXchange at PXD053016. Other data generated in this study are available within the article and its supplementary data files. All reagents and data in this paper not included within the article of its supplementary data files will be shared by the corresponding author upon request.

## Abbreviations

ATR: Serine/threonine-protein kinase ATR
AEP: Asparagine endopeptidase
BC: Breast cancer
PPP1R10: Serine/threonine-protein phosphatase 1 regulatory subunit 10
Chek1: Serine/threonine-protein kinase Chk1
P53: Cellular tumor antigen p53
IDBC: Invasive Ductal Breast Carcinoma
Lamp2: Lysosome-associated membrane glycoprotein 2
Glb1: Beta-galactosidase
CtsD: Cathepsin D
MCM6: DNA replication licensing factor MCM6
MCM7: DNA replication licensing factor MCM7
NOP56: Nucleolar protein 56
NOP58: Nucleolar protein 58
HSP90AA1: Heat shock protein HSP 90-alpha
HSP90AB1: Heat shock protein HSP 90-beta
MRE11A: Double strand break repair protein MRE11
TNBC: Triple Negative Breast Cancer
PTW-PP1: PPP1R10/TOX4/WDR82-Protein Phosphatase 1 Complex
COAD: Colon Adenocarcinoma
GBM: Glioblastoma
HNSC: Head and Neck Squamous cell Carcinoma
UVM: Uveal Melanoma
STAD: Stomach Adenocarcinoma
TCGA: The Cancer Genome Atlas
PCPG: Pheochromocytoma and Paranglioma
READ: Rectum adenocarcinoma
TOX4: TOX high mobility group box family member 4
WDR82: WD repeat-containing protein 82
PP1: Protein phosphatase 1
