# Supplementary figures and images for: Radiotherapy resistance driven by Asparagine endopeptidase through ATR pathway modulation in breast cancer
Source: J Exp Clin Cancer Res. 2025 Feb 27;44:74. doi: 10.1186/s13046-025-03334-6 (PMC11866873; doi:10.1186/s13046-025-03334-6)

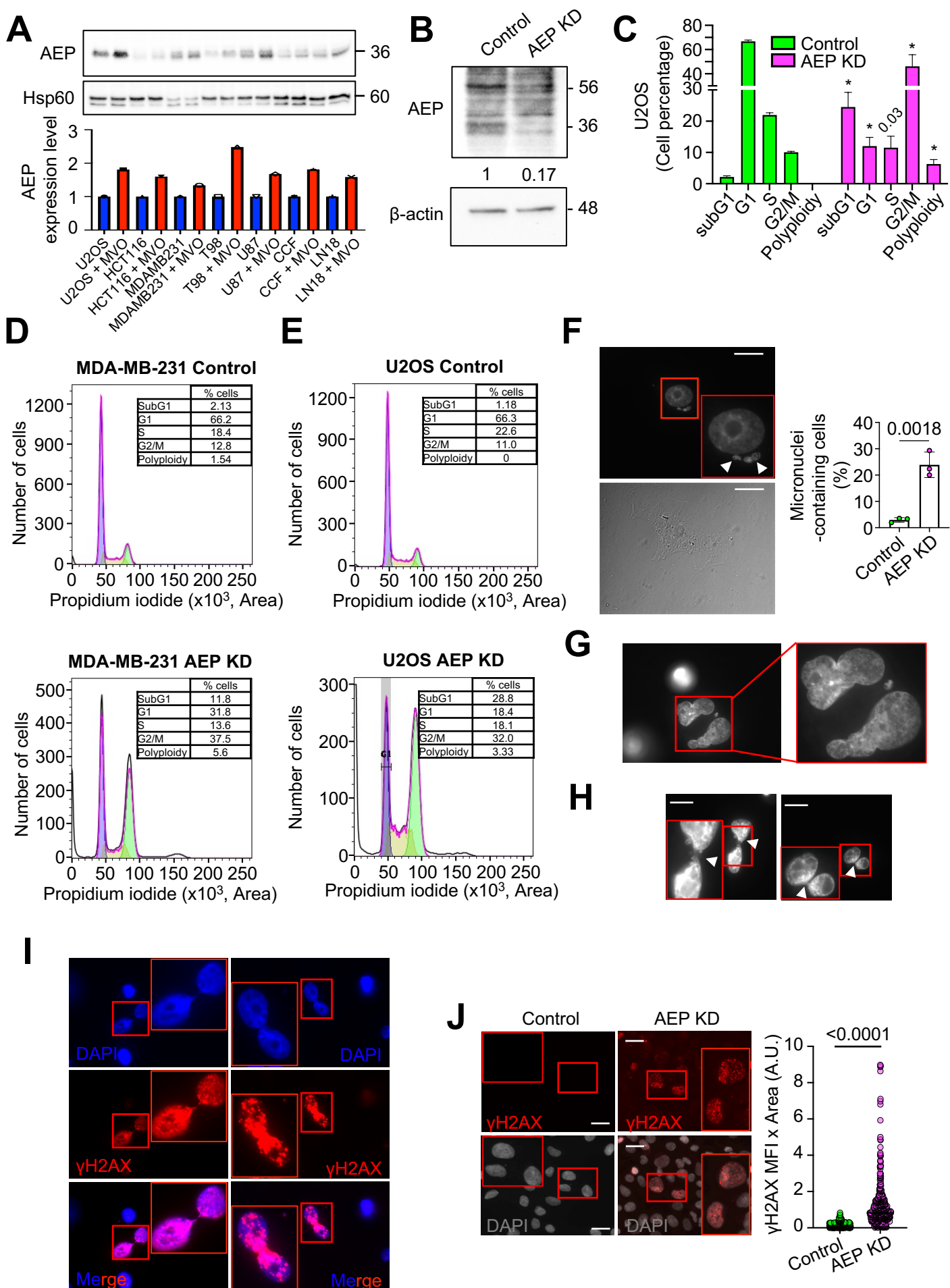

Supplementary Figure 1

Supplement: Supplementary file 1 — Additional file 1. Supplementary Figure 1. AEP deficiency in cancer cells leads to cell cycle arrest and cell death. (A) Immunoblot of AEP in different cancer cell lines untreated or treated with 50 μM MVO for 24 h, alongside quantitation. (B) Immunoblot showing the shRNA-mediated AEP knock-down in U2OS cells. (C) Cell cycle analyses of control and AEP shRNA transduced U2OS cells. Data represents average ± SD of 4 independent, biological replicas. * p value <0.01. (D) Representative image of the cell cycle profile of control (upper panel) compared to AEP KD (lower panel) MDA-MB-231 cells indicating the percentage of cells in the different phases of the cell cycle. (E) Representative image of the cell cycle profile of control (upper panel) compared to AEP KD (lower panel) U2OS cells indicating the percentage of cells in the different phases of the cell cycle. (F) Micrographs showing the presence of micronuclei in U2OS cells upon shRNA-mediated AEP KD alongside quantitation. Quantitation represents the average ± SD of three independent experiments, each one including more than 100 cell. Size bar = 27um. (G) Micrographs showing examples of polyploid cells in shRNA-mediated AEP KD U2OS cells. (H) Micrographs showing internuclear DNA bridges in shRNA-mediated AEP KD U2OS cells. Arrowheads indicate DNA bridges. Size bar = 27um. (I) Micrographs showing γH2AX-positive internuclear DNA bridges in shRNA-mediated AEP KD MDA-MD-231 cells. (J) Micrographs showing anti-γH2AX staining in both control (left panels) and AEP shRNA transduced (right panels) U2OS cells alongside quantitation representing the median. (n>400 cells). Size bar = 27um. [file 13046_2025_3334_MOESM1_ESM.pdf]

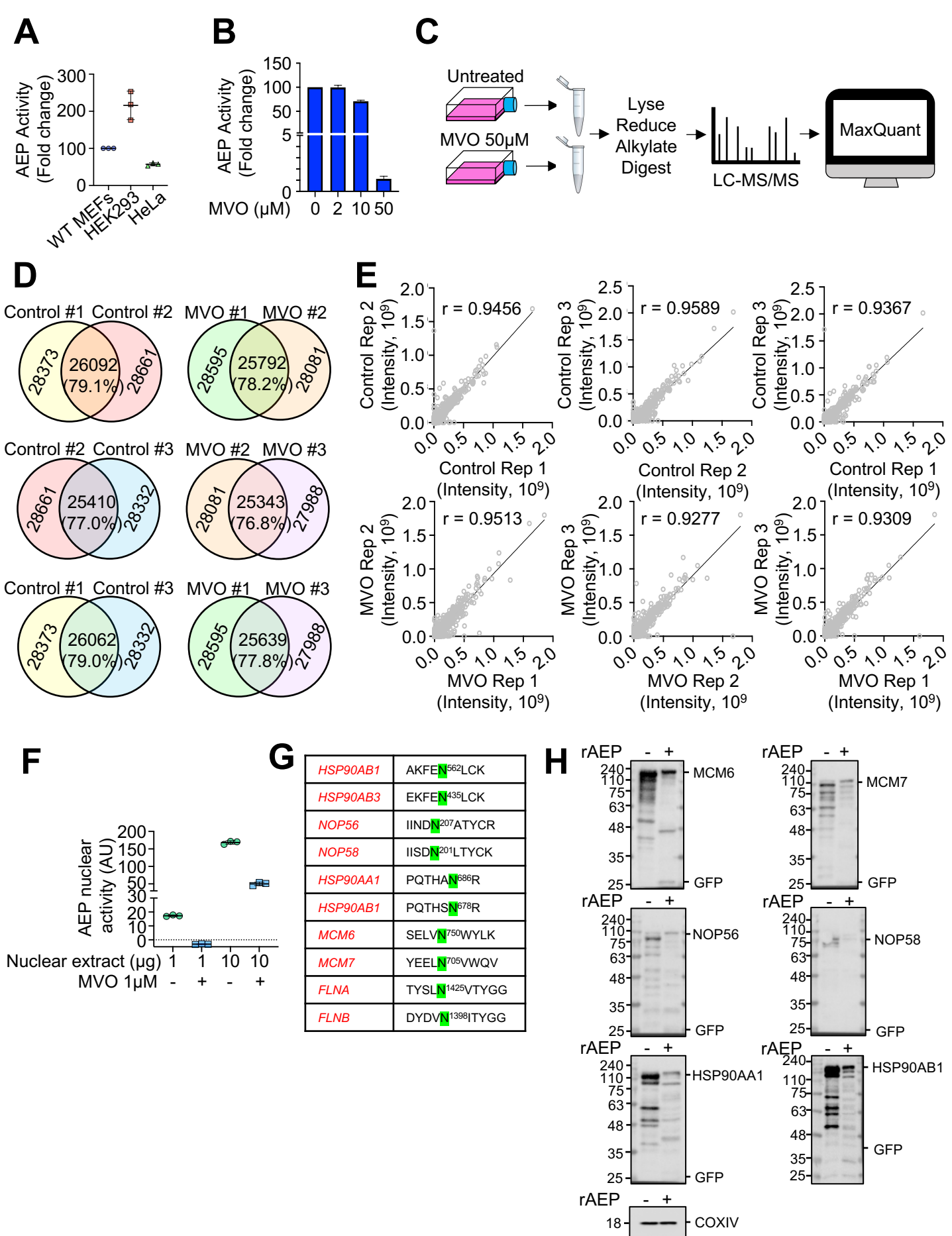

Supplementary Figure 2

Supplement: Supplementary file 2 — Additional file 2. Supplementary Figure 2. Overlap and correlation of the intensities of the peptides identified in the individual biological replicas. (A) AEP activity in MEFs, HEK293 and HeLa cells. (B) Inhibition of AEP activity in HEK293T using different concentrations of MVO26630 (0, 2, 10 or 50μM) for 16 hours. Data represents the mean ± SD of three independent, biological replicas. (C) Proteomics workflow. (D) Percentage of shared peptides identified in the individual biological replicas. (E) Correlation of the intensities of the peptides identified in the individual biological replicas. (F) AEP activity measured in nuclear extracts of HEK293 cells and the effect of the specific AEP inhibitor (MVO26630). (G) Examples of Asn/Asp containing peptides accumulating upon MVO-mediated AEP inhibition highlighting the newly identified putative cleavage sites (in green). (H) In vitro digestion of the GFP-tagged, AEP putative targets using recombinant AEP at pH7.2 for 3 hours, alongside COXIV as negative control. [file 13046_2025_3334_MOESM2_ESM.pdf]

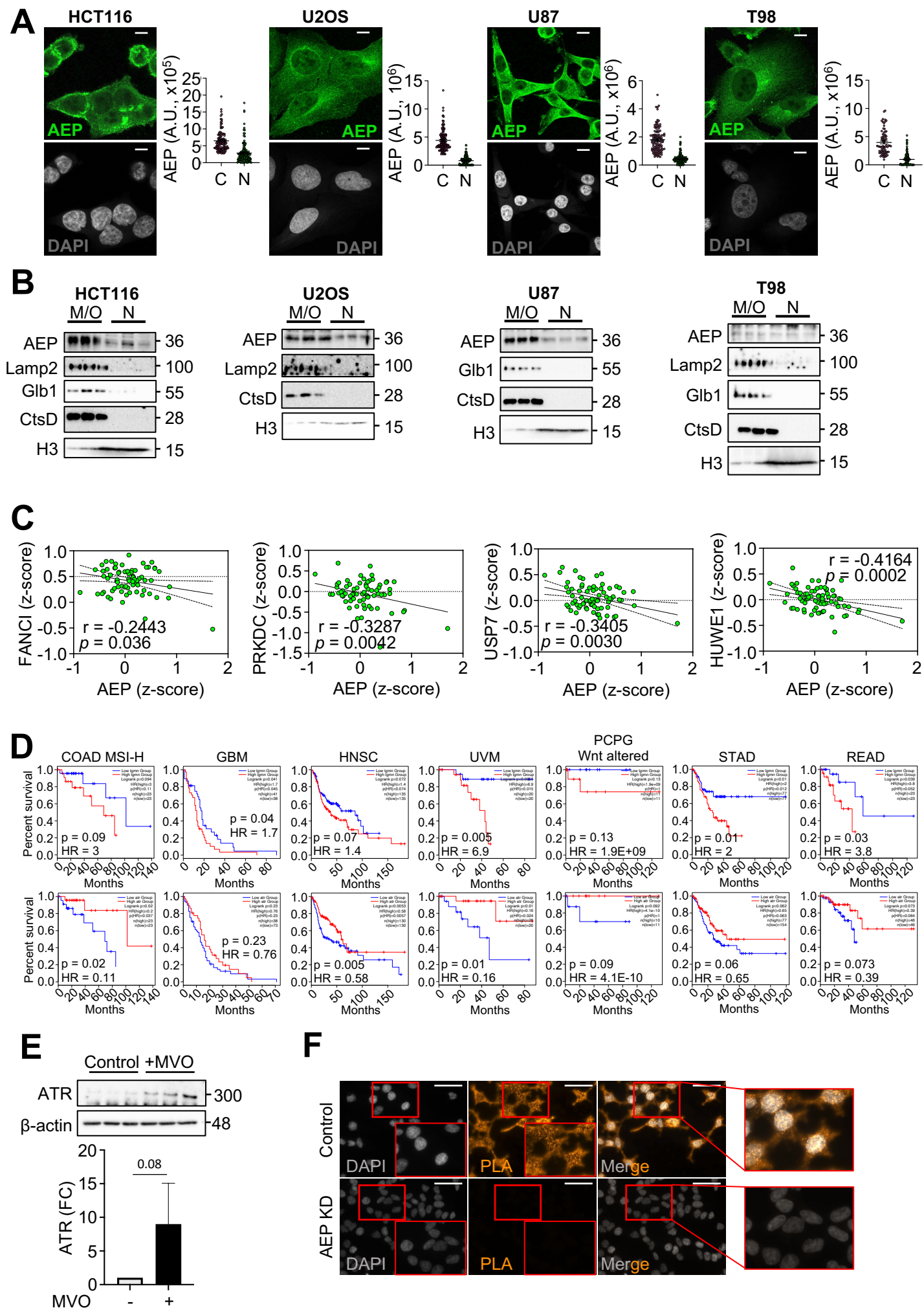

Supplementary Figure 3

Supplement: Supplementary file 3 — Additional file 3. Supplementary Figure 3. AEP regulates the levels of some of the identified targets in human cancer cells. (A) Immunofluorescence showing the subcellular localization of AEP in HCT116 (n>100 cells), U2OS (n>160 cells), U87 (n>120 cells) and T98 (n>90 cells), alongside quantitation showing for each cell line the cytoplasmic (C) and nuclear (N) intensity, corrected using a sheep IgG antibody. (Size bar=6um). (B) Immunoblot showing the nuclear (N) and membrane/organelle (M/O) localization of AEP in HCT116, U2OS, U87 and T98 cells, including Lamp2 as a lysosomal membrane marker, Glb1 and CtsD as soluble lysosomal hydrolases and H3 as a nuclear marker. (C) Correlation analysis of the protein expression levels of some of the novel AEP targets identified in our proteomic analysis vs AEP in breast cancer patients using data obtained from the TCGA database. (D) Kaplan-Meier analyses of patients of different types of cancer (Colon adenocarcinoma with high microsatellite instability (COAD MSI-H), Glioblastoma (GBM), Head and Neck Squamous cell Carcinoma (HNSC), Uveal Melanoma (UVM), Pheochromocytoma and Paranglioma with Wnt-altered (PCPG), Stomach Adenocarcinoma (STAD) and Rectum Adenocarcinoma (READ)) expressing high (red line) or low (blue line) levels of AEP (upper panels) or ATR (lower panels). (E) Immunoblot showing the effect of MVO-mediated AEP inhibition in the levels of ATR in U2OS cells alongside quantitation. Data represents the average ± SD of three independent, biological replicas. (F) Proximity ligation assay (PLA) using anti-AEP and anti-ATR in control and AEP KD MDA-MB-231 cells. [file 13046_2025_3334_MOESM3_ESM.pdf]

**A**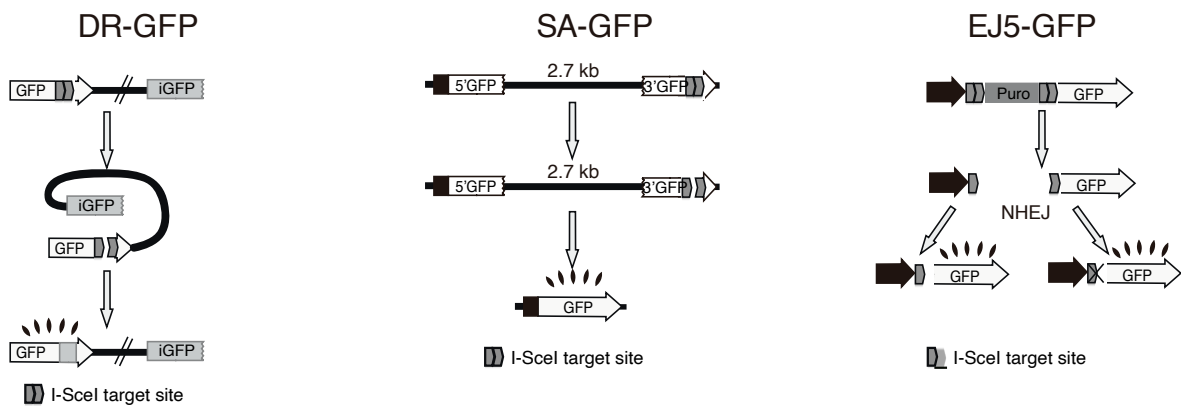**B**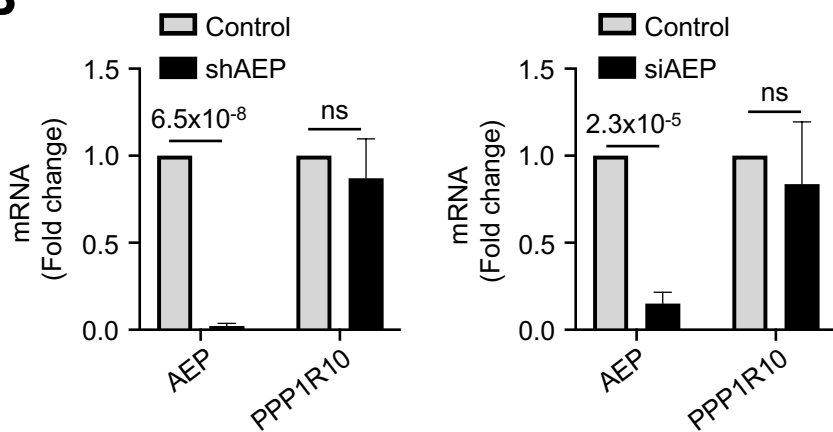**C**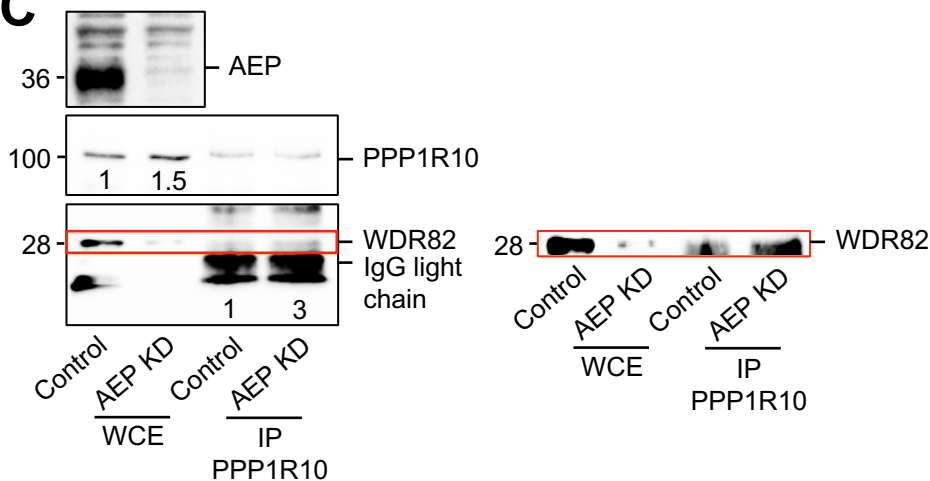**D**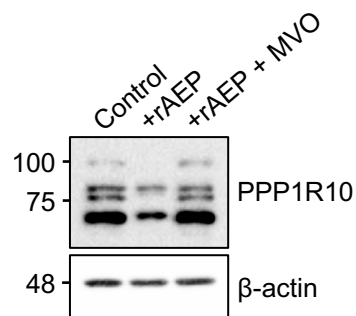**E**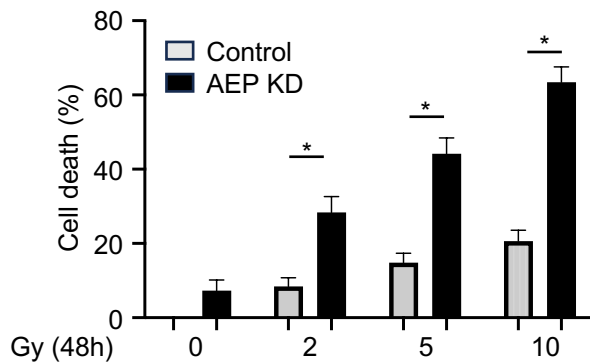**F**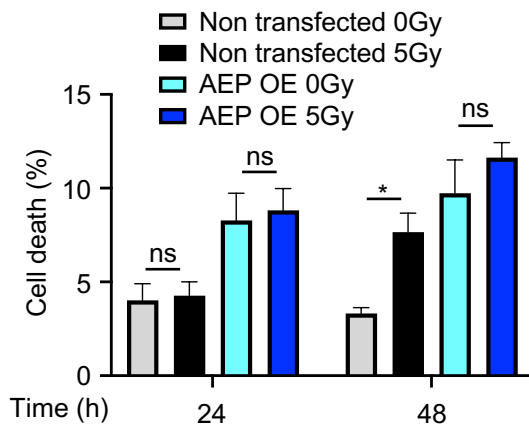

Supplement: Supplementary file 4 — Additional file 4. Supplementary Figure 4. Role of AEP in genotoxic tolerance in BC cells. (A) Schematic representation of the systems used to measure the efficiency of DNA repair by different DNA repair mechanisms: classical homologous recombination (DR-GFP), single-strand annealing (SA-GFP) and non-homologous end-joining (EJ5-GFP). (B) qPCR analyses of the mRNA expression levels of AEP and PPP1R10 in control (EV, empty vector) and AEP shRNA (left panel) or control (NT, non-targeting) and AEP siRNA KD (right panel) MDA-MB-231 cells. (C) Co-immunoprecipitation assay of WDR82 using anti-PPP1R10 in control and AEP KD MDA-MB-231 cells. In red box cropped, overexposed WDR82 immunoblot. (D) In vitro digestion of PPP1R10 using recombinant AEP at pH7.2 for 3 hours. (E) Cell death in control and AEP KD MDA-MB-231 after 48 hours irradiation using 0, 2, 5 or 10 Gy. (F) Cell death in non-transfected vs AEP overexpressing HCT116 cells after 24- or 48-hours irradiation with 0 or 5 Gy. * p<0.05, ns: no significant. [file 13046_2025_3334_MOESM4_ESM.pdf]

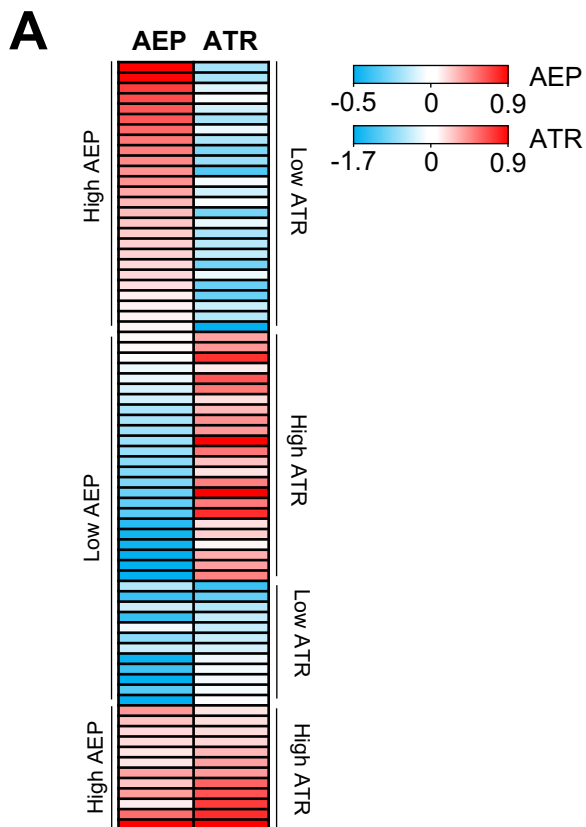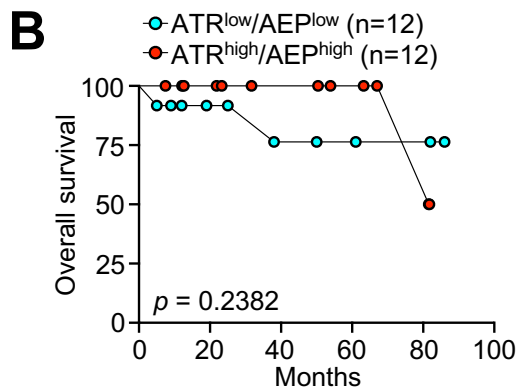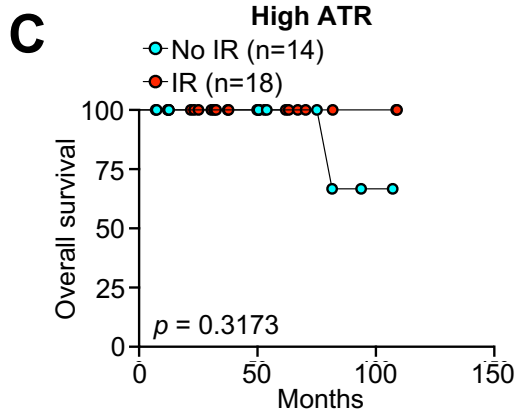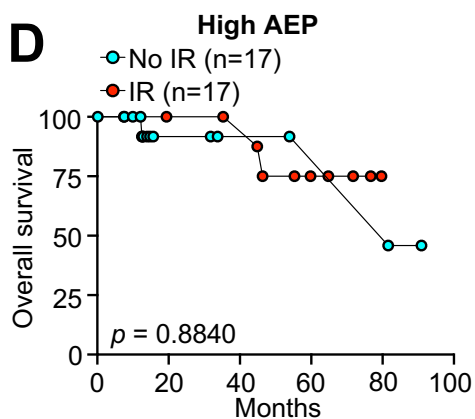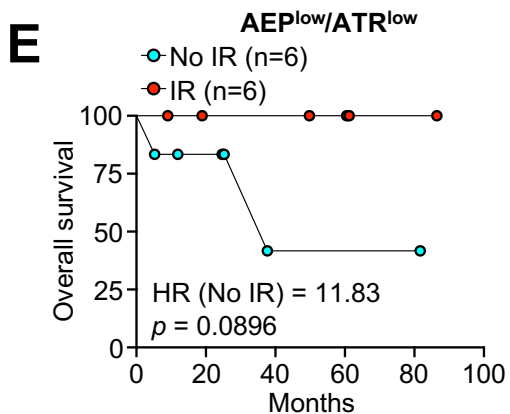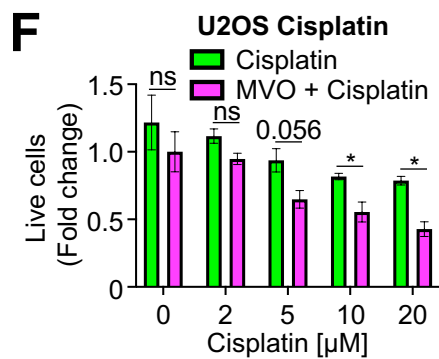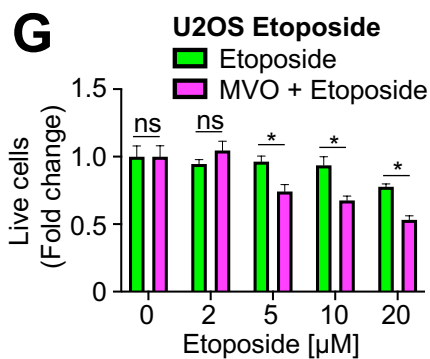

Supplementary Figure 5

Supplement: Supplementary file 5 — Additional file 5. Supplementary Figure 5. AEP inhibition sensitizes cancer cells to genotoxic stress. (A) Heatmap showing the levels of protein (AEP and ATR) expressed in breast cancer patients dividing patients in four groups (AEPhigh/ATRlow; AEPlow/ATRhigh; AEPlow/ATRlow; AEPhigh/ ATRhigh). (B) Kaplan-Meier analysis in AEPlow/ATRlow (cyan dots) or AEPhigh/ATRhigh (red dots) breast cancer patients at the protein level. (C) Kaplan-Meier analysis in breast cancer patients expressing high ATR protein levels treated (red dots) or untreated (cyan dots) with radiation. (D) Kaplan-Meier analysis in breast cancer patients expressing high protein AEP levels treated (red dots) or untreated (cyan dots) with radiation. (E) Kaplan-Meier analysis in AEPlow/ATRlow breast cancer patients treated (red dots) or untreated (cyan dots) with radiation. (F) Dose response of cisplatin in U2OS cell in the presence (magenta bars) or absence of MVO (green bars). (G) Dose response of etoposide in U2OS cell in the presence (magenta bars) or absence of MVO (green bars). ns: no significant, * p < 0.05. [file 13046_2025_3334_MOESM5_ESM.pdf]

**A**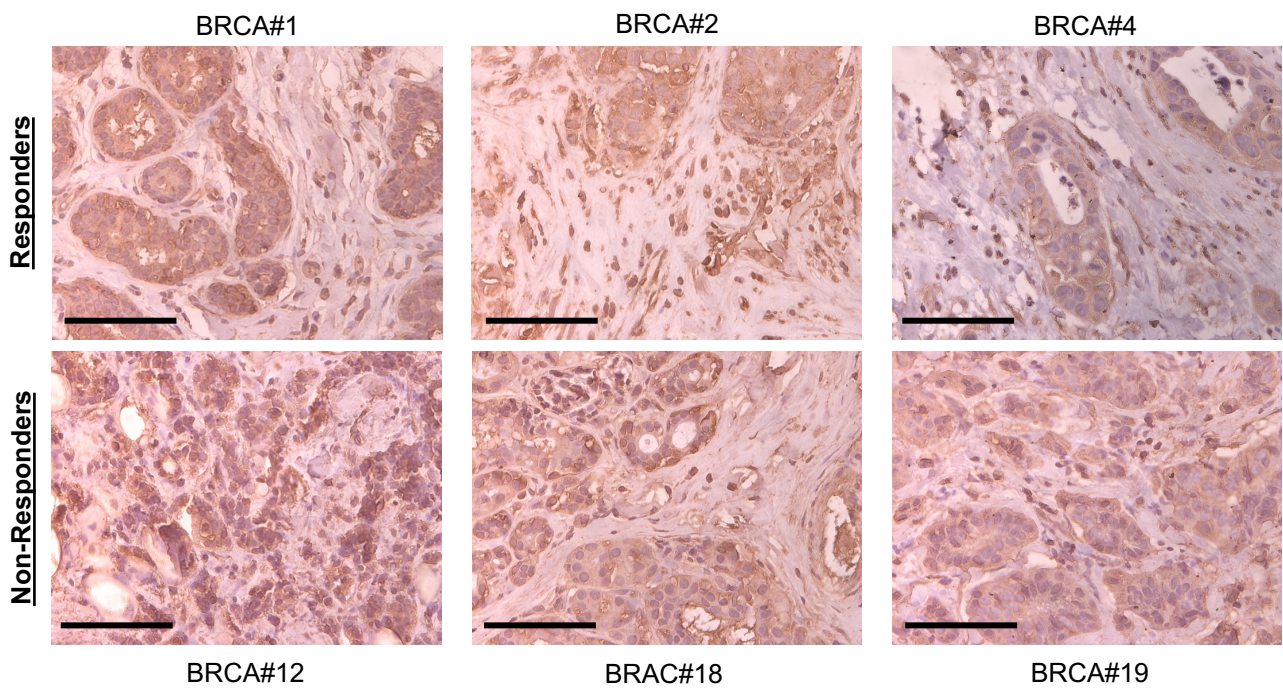**B**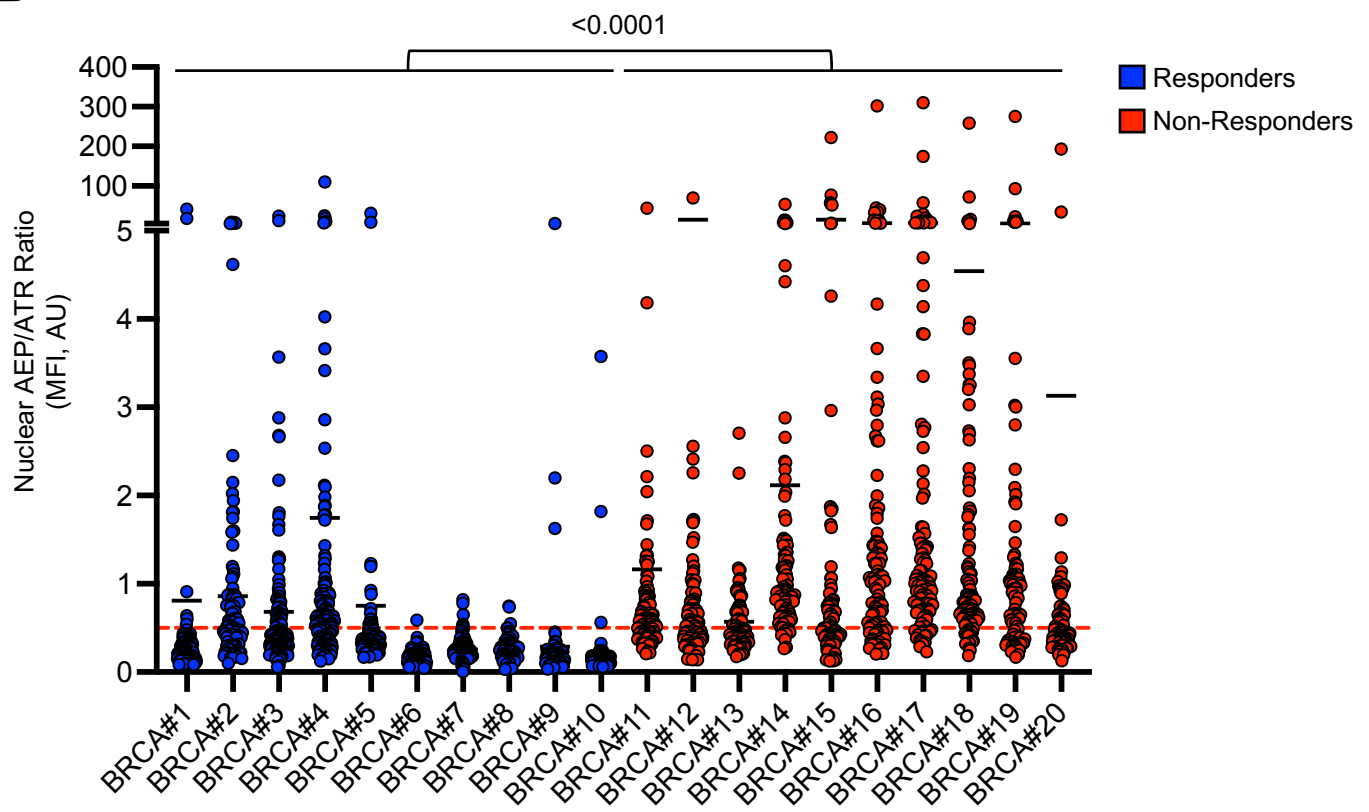

Supplement: Supplementary file 6 — Additional file 6. Supplementary Figure 6. Increased AEP/ATR levels in ductal invasive breast carcinoma non-responder patients. (A) Microscopy images (40x) of the immunohistochemical analysis of AEP expression in responder and non-responder invasive ductal breast carcinoma samples (Size bar = 40μm). (B) Nuclear AEP/ATR ratio obtained from responder (n=10, blue dots) and non-responder (n=10, red dots) invasive ductal breast carcinoma patients. Each dot represents the nuclear AEP/ATR ratio obtained for each ductal breast carcinoma cell (n>80 cells per patient). Two-way ANOVA was used to calculate the statistical significance between responder and non-responder groups. Red dotted line indicates a nuclear AEP/ATR ratio greater than 0.5. [file 13046_2025_3334_MOESM6_ESM.pdf]
